# Moderating effects of uric acid and sex on cognition and psychiatric symptoms in asymmetric Parkinson’s disease

**DOI:** 10.1186/s13293-023-00510-1

**Published:** 2023-05-04

**Authors:** Ioana Medeleine Constantin, Philippe Voruz, Julie Anne Péron

**Affiliations:** 1 Clinical and Experimental Neuropsychology Laboratory, Faculty of Psychology and Educational Sciences, University of Geneva, 40 Bd du Pont d’Arve, 1205 Geneva, Switzerland; 2 Neurology Department, Geneva University Hospitals, 4 Rue Gabrielle-Perret-Gentil, 1205 Geneva, Switzerland

**Keywords:** Parkinson’s disease, Motor symptom asymmetry, Cognition, Psychiatric symptoms, Uric acid, Three-way interaction models

## Abstract

**Background:**

Non-motor symptoms are an important early feature of Parkinson’s disease (PD), encompassing a variety of cognitive and psychiatric symptoms that seem to manifest differently depending on motor symptom asymmetry. Different factors, such as uric acid (UA) and sex, seem to influence cognitive and psychiatric expression in PD, however their interplay remains to be better understood.

**Methods:**

Participants taking part in the Parkinson’s Progression Marker Initiative were studied based on the side of motor symptom asymmetry and sex. Three-way interaction modeling was used to examine the moderating effects of sex and UA on cognitive functions and psychiatric symptoms.

**Results:**

Significant three-way interactions were highlighted at 1-year follow-up between motor symptom asymmetry, UA and sex for immediate and long-term memory in female patients exhibiting predominantly left-sided motor symptoms, and for processing speed and sleepiness in female patients exhibiting predominantly right-sided motor symptoms. No significant interactions were observed for male patients. Moreover, female patients exhibiting predominantly right-sided motor symptoms demonstrated lower serum UA concentrations and had overall better outcomes, while male patients with predominantly right-sided motor symptoms demonstrated particularly poor outcomes.

**Conclusions:**

These findings suggest that in the earliest stages of the disease, UA and sex moderate cognitive functions and psychiatric symptoms differently depending on motor asymmetry, holding important clinical implications for symptom management in patients.

**Supplementary Information:**

The online version contains supplementary material available at 10.1186/s13293-023-00510-1.

## Plain Language Summary

Parkinson’s disease is characterized by motor symptoms that usually manifest in an asymmetrical fashion. Given this motor symptom asymmetry, it is possible to distinguish patients that exhibit predominantly right-sided motor symptoms from those that exhibit predominantly left-sided motor symptoms. Patients also often develop non-motor symptoms, such as cognitive and psychiatric complaints. Recent studies have found that non-motor symptoms can manifest differently depending on motor symptom asymmetry. Furthermore, different factors, such as uric acid, a natural antioxidant in the human body, and the patient’s sex seem to influence cognitive and psychiatric manifestations, however their interplay remains to be better understood. The present study aimed to examine the interactions between motor symptom asymmetry, serum uric acid and patient’s sex on the manifestation of cognitive and psychiatric symptoms. Using regression models, it was found that at 1 year from diagnosis, uric acid and sex moderated cognitive and psychiatric symptoms differently according to motor symptom asymmetry. Indeed, female patients with predominantly left-sided motor symptoms had better memory performances with lower concentrations of serum uric acid, whereas female patients with predominantly right-sided symptoms presented better psychomotor speed and less sleepiness with higher concentrations of uric acid. Moreover, female patients with predominantly right-sided motor symptoms had overall better outcomes, while male patients with predominantly right-sided motor symptoms demonstrated particularly poor clinical outcomes. These findings suggest that in the earliest stages of the disease, uric acid and sex moderate cognitive and psychiatric symptoms differently depending on motor asymmetry, holding important clinical implications for symptom management in patients.

**Supplementary Information:**

The online version contains supplementary material available at 10.1186/s13293-023-00510-1.

## Highlights

- Significant three-way interactions were highlighted at 1-year follow-up between motor symptom asymmetry, cognitive–psychiatric symptoms and serum uric acid in female, but not male patients
- Moderation occurred for immediate memory, long-term memory, processing speed and sleepiness
- Uric acid and sex differentially moderated cognitive and psychiatric symptoms depending on the side of motor symptom asymmetry
- Female patients with right-sided symptoms demonstrated the most favorable clinical outcomes and displayed lower levels of serum uric acid
- Male patients with right-sided symptoms demonstrated the least favorable neuropsychological outcomes and presented with higher levels of serum uric acid

**Supplementary Information:**

The online version contains supplementary material available at 10.1186/s13293-023-00510-1.

## Introduction

Motor symptoms in Parkinson’s disease (PD) manifest predominantly in an asymmetrical fashion at the onset of the disease [1], with most de novo PD patients experiencing unilateral motor signs that reflect a contralateral hemispheric loss of dopamine in the substantia nigra [2]. We can thus distinguish patients that exhibit predominantly right-sided motor symptoms (RPD; left hemispheric loss of dopamine) from those that show predominantly left-sided motor symptoms (LPD; right hemispheric loss of dopamine). The clinical picture of PD patients extends however well beyond their motor deficits, encompassing a wide range of non-motor symptoms (NMS) that can manifest years before the onset of motor symptoms, becoming more predominant as the disease progresses [3].

NMS in PD encompass a variety of features, of which cognitive and psychiatric impairments [4], some of which seem to differ based on motor symptom asymmetry [5]. Indeed, from a neuropsychological standpoint, RPD patients seem to show greater cognitive impairment, notably in terms of verbal memory [6, 7], language [8], attention [9] and executive functions [10, 11]. However, LPD patients seem to be more impaired in the visuospatial domain in comparison to their RPD counterparts [12, 13]. That said, not all cognitive functions appear to be differentially impaired as a function of motor symptom asymmetry, such as inhibitory control [14]. Recent studies that have compared PD patients in early and moderate stages of the disorder have found no differences between LPD and RPD patients [15, 16]. These results were also confirmed in advanced patients in pre- and post-deep brain stimulation of the subthalamic nucleus, where only the bilateral operation restored reactive and proactive inhibitory control [17–19]. Concerning psychiatric symptomatology, different studies have highlighted higher depressive symptoms [20], anxiety [21] and emotion recognition deficits [10] in LPD patients as compared to RPD patients. Moreover, RPD patients seem to present less motor deficits than LPD patients [22]. Such differences in symptoms might be explained in terms of hemispheric vulnerability, with studies highlighting a greater vulnerability of the left hemisphere for cognitive decline in neurodegenerative diseases [23], and a functional insufficiency of the right hemisphere in psychiatric conditions, such as depression [24]. Thus, different patterns of cognitive deficits and psychiatric symptoms seem to emerge on the side of the largest hemispheric damage due to dopaminergic pathway depletion in PD. Nevertheless, discrepancies persist in the scientific literature concerning motor symptom asymmetry, cognition and psychiatric symptoms, with several studies finding reversed patterns of impairment, and others failing to report any significant differences [4, 25]. Such heterogeneity could reflect a differential effect of various underlying biological factors that interact with symptom expression in the disease.

Recent studies have linked uric acid (UA), a naturally occurring antioxidant in the human body, with a potential neuroprotective role in PD [26]. Serum UA levels seem to be significantly lower in PD, with concentrations further decreasing as the disease progresses [27]. Thus, it has been proposed that higher UA levels in PD patients are associated with a decreased risk of dementia and preserved cognitive function [26, 28]. Indeed, it has been found that baseline serum UA can be a useful marker of cognitive deficits and psychiatric symptom progression in newly diagnosed PD, with higher values corresponding to preserved attention and memory performances [28], as well as diminished symptoms of depression, anxiety [29] and fatigue [30, 31]. Interestingly, UA also seems to be associated to different aspects of motor symptoms in PD, such as motor fluctuations and motor subtypes [31–33]. Notwithstanding, results remain inconclusive, as other studies suggest an opposite effect of high serum UA on cognitive functions [34–36]. The effect of UA on disease symptoms and progression seems to be moderated by participants’ sex, possibly explaining the heterogenous findings of previous studies that didn’t consider differences between men and women [37]. Men tend to have greater serum UA concentrations than women, with higher levels of UA being associated to a lowered risk of cognitive impairment, motor fluctuations and disease progression [38]. However, no such association was effectively found in women with PD [26, 32, 39]. Rather, it has been suggested that women may be particularly more vulnerable to the vascular effects of elevated UA [37]. Other than moderating the effects of UA on cognitive outcomes and disease progression, sex-related differences also seem to be at play in the relationship between motor symptom asymmetry, cognition and psychiatric symptoms in PD. While data are scarce concerning the simultaneous effects of asymmetry and sex on cognition, Davidsdottir et al. [40] found that male RPD patients exhibited higher visuospatial impairment compared to female RPD patients, with no differences noted between male and female LPD patients. Also, Bentivoglio et al. [41] highlighted better performances in language tasks for female PD patients, independently of the onset side of motor symptoms.

In summary, different clinical factors, such as motor symptom asymmetry, UA and sex, seem to influence cognitive and psychiatric manifestations in PD; however, no study has yet examined the interactive effects of these factors. Hence, the present study aims to better understand the interactions between the effects of UA, sex and motor symptom asymmetry on cognition and psychiatric symptoms in early-stage PD. The following predictions were formulated based on the current state of the literature. Using three-way interaction (moderated moderation) models [42], we expected sex and UA to significantly moderate the relationship between motor symptom asymmetry and cognitive functions, as well as psychiatric symptoms. Firstly, we expected stronger moderation effects in men, with higher UA concentrations corresponding to better cognitive and psychiatric outcomes [38, 43]. Secondly, we expected lesser moderating effects in women, or even an inverse association between UA and cognitive functions as well as psychiatric symptoms, with female patients having better preserved outcomes in the presence of lowered levels of UA [37]. Finally, the effects of sex and UA when taking in consideration motor symptom asymmetry were explored.

## Methods

### Participants

The analyzed data were obtained from the Parkinson’s Progression Marker Initiative (PPMI), an international and multicenter longitudinal study launched in 2010, aiming to identify progression biomarkers of PD [44]. The study officially enrolled 426 de novo, untreated PD patients [413 asymmetric motor symptom onset, 13 symmetric motor symptom onset (not included in this study) and 196 healthy controls (HC)]. Baseline, 1-year, 3-year and 5-year follow-up measurements were retained for the present paper. Inclusion criteria for participants was being aged above 30 years old; having newly diagnosed PD (2 years or less); being untreated with PD medication; having two of the following symptoms: resting tremor, bradykinesia and rigidity, or either asymmetric resting tremor, or asymmetric bradykinesia; having an imaging confirmation of a dopamine transporter deficit. Sex was defined as genetically confirmed sex, which was consistent with gender for all participants [45]. The asymmetry of motor symptoms was determined by a clinician at the time of diagnosis, based on the lateralized items of the Movement Disorders Society Unified Parkinson’s Disease Rating Scale (MDS-UPDRS) scale [46, 47].

A total of six groups were defined for this study: (1) PD patients with predominantly left-sided motor symptoms (LPD) at the onset of the disease, divided in male (LPDm; *n* = 105) and female (LPDf; *n* = 74) subgroups; (2) PD patients with predominantly right-sided motor symptoms (RPD) at the onset of the disease, divided in male (RPDm; *n* = 163) and female (RPDf; *n* = 71) subgroups; and (3) HC, both male (HCm; *n* = 126) and female (HCf; *n* = 70), with no clinically significant neurological disorders (see Table 1). No significant differences were observed between the 6 subgroups for sociodemographic outcomes, except for race [with a majority of White individuals in all subgroups (> 90%); results seem to be driven by a lesser diversity in the RPDm group] and initial PD symptomatology (LPDf; LPDm; RPDf; RPDm) (see Table 1).

**Table 1:** Sociodemographic and clinical variables at the baseline for each group (LPDf; LPDm; RPDf; RPDm; HCf; HCm)

	LPDf (*n* = 74)	LPDm (*n* = 105)	RPDf (*n* = 71)	RPDm (*n* = 163)	HCf (*n* = 70)	HCm (*n* = 126)	K–W/Chi^2^
Age in years (mean ± SD)	60.16 (± 10.71)	60.53 (± 9.55)	61.13 (± 8.35)	62.79 (± 9.75)	59.37 (± 11.70)	61.62 (± 10.92)	0.103
Education in years (mean ± SD)	15.19 (± 3.20)	15.58 (± 2.80)	15.31 (± 3.02)	15.74 (± 3.00)	15.49 (± 2.72)	16.35 (± 2.95)	0.088
Race [White; Black; Asian; other] (in %)	91.89; 0; 2.70; 5.41	91.43; 2.86; 3.81; 1.90	90.14; 2.81; 1.41; 5.63	95.71; 0; 0; 4.29	91.43; 4.29; 1.43; 2.86	92.86; 5.56; 0; 1.59	0.031
Age of onset in years (mean ± SD)	58.13 (± 10.94)	58.61 (± 9.90)	58.87 (± 8.73)	60.84 (± 9.93)	–	–	0.070
Age at diagnostic in years (mean ± SD)	59.58 (± 10.68)	60.05 (± 9.60)	60.52 (± 8.36)	62.22 (± 9.68)	–	–	0.078
Initial symptom (at diagnosis)							
Resting tremor	No: 17.57%	No: 26.67%	No: 25.35%	No: 17.79%	No: 100.00%	No: 100.00%	0.230
	Yes: 82.43%	Yes: 73.33%	Yes: 74.65%	Yes: 82.21%	Yes: 0.00%	Yes: 0.00%	
Rigidity	No: 29.73%	No: 19.05%	No: 32.39%	No: 20.86%	No: 100.00%	No: 100.00%	0.095
	Yes: 70.27%	Yes: 80.95%	Yes: 67.61%	Yes: 79.14%	Yes: 0.00%	Yes: 0.00%	
Bradykinesia	No: 16.22%	No: 11.43%	No: 22.54%	No: 20.25%	No: 100.00%	No: 100.00%	0.186
	Yes: 83.78%	Yes: 88.57%	Yes: 77.46%	Yes: 79.75%	Yes: 0.00%	Yes: 0.00%	
Postural instability	No: 91.89%	No: 92.38%	No: 94.37%	No: 94.48%	No: 100.00%	No: 100.00%	0.833
	Yes: 8.11%	Yes: 7.62%	Yes: 5.63%	Yes: 5.52%	Yes: 0.00%	Yes: 0.00%	
Other	No: 79.73%	No: 87.62%	No: 81.69%	No: 84.66%	No: 100.00%	No: 100.00%	0.500
	Yes: 20.27%	Yes: 12.38%	Yes: 18.31%	Yes: 15.34%	Yes: 0.00%	Yes: 0.00%	

*f* female, *HC* healthy controls, *LPD* patients with Parkinson’s disease (PD) who exhibit predominantly left-sided motor symptoms, *m* male, *RPD* patients with PD who exhibit predominantly right-sided motor symptoms, *K–W* Kruskal–Wallis test, *M–W* Mann–Whitney U test, *SD* standard deviation

### Ethics

The PPMI study is registered with ClinicalTrials.gov (NCT01141023). All participating sites received approval from an ethical standards committee on human experimentation prior to study initiation. The study was conducted in accordance with the Declaration of Helsinki and the Good Clinical Practice guidelines following approval of the local ethics committees of the participating sites. Written informed consent for research was obtained from all individuals taking part in the study. The data used in the preparation of this article were obtained from the PPMI open access database. For more information regarding the study’s protocol, research documentations and standard operating procedures, including ethical submissions and approvals, please visit https://www.ppmi-info.org/study-design/research-documents-and-sops.

### Measured outcomes

Participants underwent comprehensive clinical, imaging and biosampling assessments. Regarding clinical aspects, the MDS-UPDRS [48] and Hoehn and Yahr scale [49] were used to evaluate motor aspects, and Epworth Sleepiness Scale (ESS) [50] was used to measure daytime sleepiness. Among cognitive assessments, the Montreal Cognitive Assessment (MoCA) [51], Hopkins Verbal Learning Test (HVLT) [52], Benton Judgment of Line Orientation Test (BJLOT) [53], semantic fluency [54] and Symbol Digit Modalities Test (SDMT) [55]were used. The Geriatric Depression Scale (GDS) [56] and State-Trait Anxiety Inventory (STAI) [57] were used to assess psychiatric symptoms. Blood sampling was used to measure plasma levels of UA at baseline and at each 12-month follow-up visit. Forty milliliters of venous whole blood was collected and sent to a central laboratory for analysis, thus guaranteeing identical analysis methods and consistent normal ranges.

### Data analysis

**Fig. 1:**
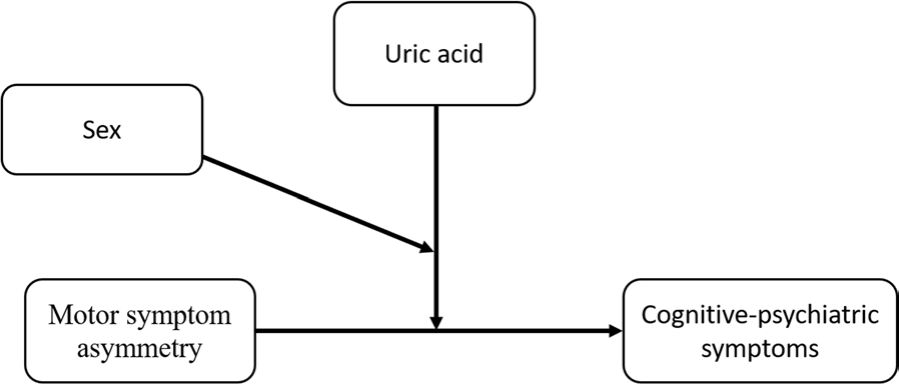
Conceptual model of a three-way interaction between motor symptom asymmetry, UA and sex. A conceptual representation of three-way interaction (moderated moderation) effects of UA and sex on the relationship between motor symptom asymmetry and cognitive–psychiatric symptoms, extracted from PROCESS macro for SPSS (Model 3)

In order to assess the relationships between UA, sex and motor symptom asymmetry on cognitive and psychiatric outcomes, we tested a conceptual three-way interaction model, also known as moderated moderation model, using the Hayes PROCESS macro [42]. The three-way moderation model included motor symptom asymmetry (multicategorical indicator coding system for RPD and LPD) as the predictor variable, cognitive and psychiatric symptoms as outcome variables, UA as the primary moderator (M) and sex as the secondary moderator (W; Fig. 1). This model was tested for each outcome, namely cognitive (MoCA, HVLT immediate and delayed recall, BJLOT, SDMT and semantic fluency scores) and psychiatric (GDS and STAI scores) variables, at each timepoint. Given the impact of age, racial background, levodopa medication and education on individuals’ UA serum concentrations and NMS [28, 58, 59], we adjusted for these three variables by including them as covariates in the models. The Davidson–Mackinnon estimator of heteroskedasticity-consistent standard error was used in order to reduce the possible effects of heteroskedasticity on the inference of regression estimates [60]. All data analyses were conducted in SPSS 26.

## Results

### Moderated moderation in the relationship between motor symptom asymmetry and cognitive and psychiatric symptoms

**Fig. 2:**
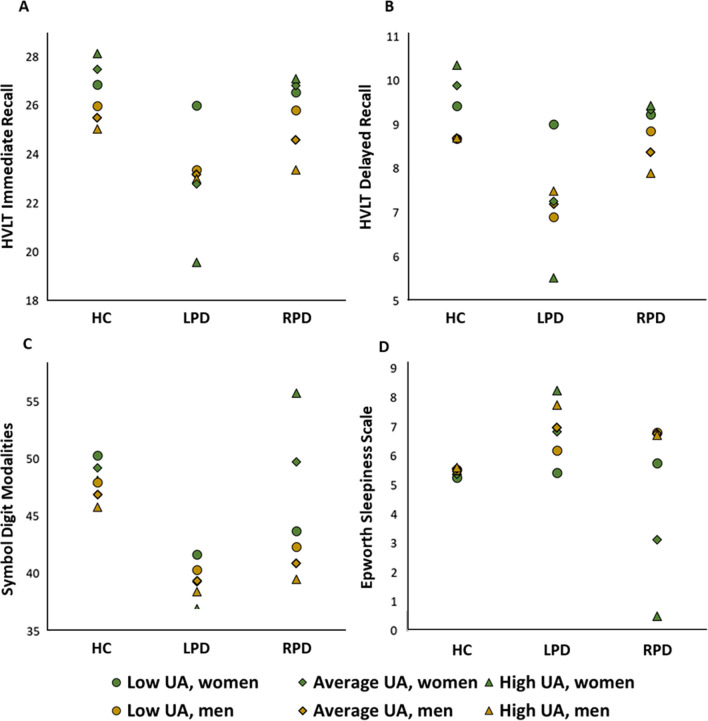
Three-way interaction plot of motor symptom asymmetry, UA and sex on cognitive and psychiatric outcomes. Significant three-way interactions were noted for immediate memory (**A**), delayed memory (**B**), processing speed (**C**) and sleepiness (**D**). Analyses were performed using the PROCESS macro for SPSS (Model 3). Model includes the following covariables: age, education, levodopa medication and racial background. *HC* healthy controls, *HVLT* Hopkins Verbal Learning Test, *LPD* patients with Parkinson’s disease (PD) who exhibit predominantly left-sided motor symptoms, *RPD* patients with PD who exhibit predominantly right-sided motor symptoms, *UA* uric acid

The three-way interaction models yielded interesting results regarding the moderating effects of UA and sex on the relationship between motor symptom asymmetry, clinically measured using the MDS-UPDRS III scores at disease onset, and cognitive and psychiatric symptoms. A total of four models showed a statistically significant interaction between motor symptom asymmetry, UA and sex at 1-year follow-up (see Table 2). Model 1 showed a statistically significant interaction between the LPD group, UA levels and sex on immediate memory scores. The overall model accounted for 24.46% of the total variance of immediate memory recall scores [*F*(15, 372) = 8.44, *p* < 0.001], with the interaction itself accounting for 1.63% of the total variance. Model 2 also displayed a significant interaction between the LPD group, UA levels and sex on delayed memory scores. The overall model accounted for 21.73% of the total variance [*F*(15, 372) = 9.11, *p* < 0.001], with the three-way interaction accounting in itself for 2.07% of the total variance. These two first models demonstrate that LPDf patients seem to be more affected by UA variability than their male counterparts, showing higher memory scores in the presence of lower levels of UA (see Fig. 2A, B). Model 3, on the other hand, demonstrated a statistically significant interaction between the RPD group, UA and sex for processing speed scores. The overall model accounted for 38.89% of the total variance of SDMT scores [*F*(15, 372) = 13.61, *p* < 0.001], with the three-way interaction accounting in itself for 0.87% of the total variance. Interestingly, this model revealed better processing speed scores in the presence of higher UA concentrations in RPDf patients (see Fig. 2C). Finally, model 4 showed a statistically significant interaction between the RPD group, UA levels and sex on sleepiness. The overall model accounted for 9.09% of the total variance of ESS scores [*F*(15, 372) = 2.79, *p* < 0.001], with solely the three-way interaction accounting for 1.00% of the total variance. This model revealed that RPDf patients seem to show lower daytime sleepiness in the presence of higher UA levels (see Fig. 2D). No significant three-way interactions were found for years 3 and 5. Moreover, PD male patients did not seem to exhibit any effect of UA variability on cognitive and psychiatric measurements.

**Table 2:** Moderated moderation models showing three-way interactions between motor symptom asymmetry, UA and sex on cognitive and psychiatric outcomes at 1-year follow-up

	*B*	SE	95% CI	
Model 1: three-way interactions on HVLT immediate recall				
LPD × UA	0.060	0.031	− 0.002	0.122
RPD × UA	− 0.016	0.034	− 0.083	0.051
LPD × sex	− 2.40	1.99	− 6.31	1.52
RPD × sex	0.25	2.34	− 4.36	4.86
UA × sex	0.015	0.010	− 0.004	0.034
LPD × UA × sex	− 0.056	0.026	− 0.107	− 0.005*
RPD × UA × sex	0.005	0.029	− 0.051	0.062
Model 2: three-way interactions on HVLT delayed recall				
LPD × UA	0.038	0.015	0.009	0.067*
RPD × UA	− 0.008	0.018	− 0.044	0.028
LPD × sex	− 1.137	0.886	− 2.88	0.605
RPD × sex	− 0.244	1.11	− 2.44	1.95
UA × sex	0.006	0.005	− 0.003	0.016
LPD × UA × sex	− 0.034	0.011	− 0.0553	− 0.012*
RPD × UA × sex	0.002	0.016	− 0.030	0.033
Model 3: three-way interactions on SDMT				
LPD × UA	0.021	0.060	− 0.097	0.138
RPD × UA	− 0.106	0.060	− 0.224	0.013
LPD × sex	− 2.43	3.30	− 8.93	4.06
RPD × sex	6.49	3.83	− 1.05	14.03
UA × sex	0.000	0.027	− 0.053	0.053
LPD × UA × sex	− 0.019	0.043	− 0.105	0.067
RPD × UA × sex	0.101	0.047	0.009	0.193*
Model 4: three-way interactions on ESS				
LPD × UA	0.003	0.026	− 0.048	0.053
RPD × UA	0.035	0.022	− 0.008	0.078
LPD × sex	0.054	1.78	− 3.45	3.56
RPD × sex	− 3.45	1.37	− 6.15	− 0.748*
UA × sex	0.001	0.008	− 0.015	0.017
LPD × UA × sex	0.007	0.022	− 0.036	0.050
RPD × UA × sex	− 0.036	0.016	− 0.067	− 0.005*

Model includes the following demographic control variables: age, education, levodopa medication and racial background

*B* Unstandardized regression coefficient, *CI* Confidence Intervals, *ESS* Epworth Sleepiness Scale, *HVLT* Hopkins Verbal Learning Test, *LPD* patients with Parkinson’s disease (PD) who exhibit predominantly left-sided motor symptoms, *RPD* patients with PD who exhibit predominantly right-sided motor symptoms,* SE* standard error of the regression, *SDMT* Symbol Digit Modalities test, *UA* uric acid

Statistically significant relationships where *p* < 0.05 are reported with an asterisk

While supporting our hypothesis of significant moderating effects of sex and UA on the relationship between motor symptom asymmetry and neuropsychiatric outcomes, these results do not go in the direction of our first prediction of a stronger moderation effect in men. Indeed, no significant and positive associations between UA concentrations and neuropsychological performances were found in male RPD and LPD patients. The results do however comfort our second prediction of an inverse association between UA and cognitive performances in female patients, with better outcomes noted in the presence of lower UA levels for the LPDf group. The simultaneous interaction between motor symptom asymmetry, sex and UA was explored, showing an inverse moderating effect of UA depending on the side of motor symptomatology and the type of NMS in women, with RPDf patients showing less sleepiness and improved processing speed in the presence of higher UA concentrations.

### Cognitive and psychiatric outcomes according to motor symptom asymmetry and sex

Secondary analyses were conducted to better characterize the profile of each subgroup. Inter-group and intra-group comparisons with respect to motor asymmetry and sex were carried out for cognitive scores (Additional file 1: Tables S1.1, S1.2 and S1.3, Fig. S1), psychiatric scores (Additional file 1: Tables S2.1, S2.2 and S2.3, Fig. S2), as well as motor severity symptoms alongside serum UA and Levodopa Daily medication (Additional file 1: Tables S3.1, S3.2 and S3.3, Fig. S3).

In brief, female patients had overall better cognitive performances compared to their male counterparts, for the MoCA (*p* < 0.05), HVLT immediate and delayed recall (*p* < 0.02), BJLOT (*p* < 0.008), SDMT (*p* < 0.03) and semantic fluency (*p* < 0.003). More so, the RPDf subgroup demonstrated better cognitive functioning, notably for the MoCA (*p* = 0.002), HVLT immediate and delayed recall (*p* < 0.023) and semantic fluency (*p* < 0.03), when compared to HC and their male counterparts. They also showed the least decline over time (noted only for the BJLOT at Year 1, *p* < 0.007). Similar patterns of results were observed for psychiatric measures, with the RPDf group demonstrating lesser symptoms and the least decline over time when compared to other groups. It is noteworthy that RPDf patients exhibited the lowest concentrations of UA at certain timepoints (*p* < 0.04). When compared to the rest of the subgroups, RPDm patients were most affected on multiple domains, such as global efficiency (*p* < 0.032), short and long-term memory (*p* < 0.03) as well as processing speed (*p* < 0.012). The RPDm group also displayed the most important decline over time in terms of sleepiness (*p* < 0.001), depressive symptoms (*p* < 0.004) and sleep disturbances (*p* < 0.001). Finally, significant negative correlations were highlighted between the scores of the ESS and the SDMT for the whole sample and for each patient subgroup, except for LPDf patients (see Additional file 1: Table S4). Further information can be found in the Additional file 1.

## Discussion

The present study aimed to better understand the relationships between UA, sex and motor symptom asymmetry at disease onset on cognitive functions and psychiatric symptoms in early-stage PD patients. To this end, we performed three-way interaction models using data from the Parkinson’s Progression Marker Initiative. We expected sex and UA to significantly moderate the relationship between motor symptom asymmetry and cognitive–psychiatric symptoms, with stronger moderation effects in men, where higher UA concentrations would correspond to better cognitive and psychiatric outcomes. Conversely, we expected lesser moderating effects in women, or even an inverse association between UA and cognitive–psychiatric symptoms, with female patients having preserved outcomes in the presence of lowered UA levels. Finally, the effects of sex and UA when taking in consideration motor symptom asymmetry were explored. Results of our three-way interactions partially confirmed our predictions regarding certain cognitive functions and psychiatric symptoms at 1-year follow-up, offering strong support for a significant moderation of UA and sex on the relationship between motor symptom asymmetry and cognitive-psychiatric symptoms. Indeed, as expected, significant three-way interactions were found for the LPD and RPD female subgroups. For the LPDf subgroup, higher short- and long-term memory performances were associated to lower concentrations of UA, while for the RPDf subgroup, higher daytime sleepiness and slower processing speed were associated to lower concentrations of UA. Finally, the male subgroups did not seem to beneficiate from the modulating effects of UA.

Three patterns of results seem to stand out from the current findings. First, the results of our three-way interactions corroborate previous findings of a deleterious effect of elevated UA concentrations on certain cognitive functions, such as episodic memory, in women [37]. In this regard, lower serum UA could be an indicator of an endogenic capacity to cope with PD-related cerebral oxidative stress induced by multiple neuropathological factors, such as protein misfolding, mitochondrial dysfunction, excitotoxicity, etc. [61]. Decreased UA serum concentrations in early PD patients would thus reflect an efficient mobilization of peripheral antioxidant resources to the brain. This phenomenon has also been observed in post-traumatic brain injury studies showing that decreased UA serum levels concurred with increased UA concentrations in damaged brain tissues [62].

Second, the association between UA, sex and cognitive and psychiatric outcomes seems to differ depending on the side of motor symptomatology and on the nature of the evaluated processes. For instance, in contrast to the associations found between lower UA levels and better cognitive performance in LPDf patients, RPDf patients with lower UA levels presented higher daytime sleepiness. Multiple studies have found lower serum UA levels to be associated with increased fatigue and sleepiness in PD [30, 31] (for review, see [63]), and in other clinical populations (e.g., stroke patients [64]). Moreover, previous findings have also linked lowered levels of UA with higher psychiatric manifestations in early PD [29]. Interestingly, secondary analyses highlighted significant negative correlations between higher daytime sleepiness and reduced processing speed in the RPDf group but not in the LPDf group, indicating that increased fatigue might explain the lower performances on the SDMT in the presence of decreased serum UA levels solely for the former group. Thus, while lower serum UA seems to be beneficial for preserving higher cognitive functions (such as memory) in female patients with predominantly left-sided symptoms, it might predispose to higher sleepiness, and thus reduced processing speed, in female patients with predominantly right-sided symptoms.

Third, and finally, certain cognitive functions and psychiatric assessments were not significantly associated with UA, sex nor motor symptom asymmetry. This could be explained by the fact that a substantial part of cognitive and affective processes is underpinned by bilateral neuronal networks, implicating thus a potential influence of compensatory processes at the beginning of the pathology [65]. Moreover, complex interactions with other biomarkers (e.g., total-tau; α-synuclein; Aβ42), for which distinct profiles have been recently highlighted between LPD and RPD patients [66, 67] might explain these results. Therefore, there seem to be distinct mechanisms through which UA moderates cognitive and psychiatric symptoms in early PD, notably depending on sex and motor symptom asymmetry.

Secondary analyses (detailed in Additional file 1) allowed us to further understand the clinical profiles of the subgroups. In essence, the RPDf group showed the least cognitive and psychiatric symptoms, while LPDf patients seemed more impaired in terms of cognitive functions and displayed higher psychiatric symptoms over time. Both male patient groups had overall lower cognitive performances, not to mention more significant pejoration in time, especially for the RPDm subgroup. These results comfort previous studies that found greater cognitive impairment in PD men than women [68], as well as recent longitudinal studies in early and advanced asymmetric PD that found a greater progression of cognitive impairment in RPD, specifically for global cognitive efficiency and memory function, alongside higher levels of apathy, suggesting a potential risk factor for dementia associated with PD. Conversely, results for LPD patients have revealed higher psychiatric symptoms, such as depression and anxiety, as well as emotional recognition disorders [10, 66, 69–71]. One explanation could reside in sex-related differences in hemispheric lateralization that might further allow to understand the observed cognitive and psychiatric discrepancies between subgroups. Indeed, studies tend to show greater hemispheric lateralization in men than women, with men demonstrating greater rightward connectivity, and women showing greater leftward connectivity [57]. Greater rightward lateralization in men could translate in weaker leftward plasticity, leaving the RPDm subgroup more vulnerable to the effects of pathological aging [58]. This is of particular importance as the left hemisphere has been shown to present an important vulnerability in neurodegenerative diseases, such as PD [17, 59]. On the contrary, lesser hemispheric lateralization in female PD patients, alongside a higher leftward cognitive reserve, would enable RPDf patients to better compensate for the effects of their prevalent left-hemispheric neurodegeneration.

Several limitations are noted for the present study. First, while we aimed to control for pertinent confounding variables in order to improve robustness, multiple other factors can influence purine metabolism and UA synthesis, notably diet and sex hormones [72]. It would be pertinent for future studies to better understand the interactions between sex-specific hormones, UA and alimentation in PD. Second, the models were established by yearly follow-up, transversally limiting data interpretation. It would be interesting for future studies to investigate the effects of sex and UA on the evolution of NMS using longitudinal approaches, such as generalized estimating equation modeling. Third, limitations regarding cognitive and psychiatric symptom testing should be mentioned. Notably, not all neuropsychological functions were assessed, such as inhibitory control or language. Also, assessments of psychiatric symptoms were done using only questionnaires. Fourthly, quality of life, an outcome that might also be influenced by sex and motor symptom asymmetry [69], was not assessed. Finally, as the association between serum UA and neurologic dysfunction appears to be non-linear (possibly U-shaped [34]), the changes in the dynamic relationship between UA and NMS throughout the course of the disease merit further investigation.

## Perspectives and significance

In summary, three-way interaction modeling was used in the present work, highlighting significant moderation of serum UA and sex on cognitive and psychiatric symptoms, depending on motor symptom asymmetry. Indeed, while lower serum UA appears to be associated with better memory performances in LPDf patients, it might predispose to higher sleepiness and reduced psychomotor speed in RPDf patients. Male patients, however, did not seem to beneficiate from the modulating effects of UA. Furthermore, RPDf patients displayed overall the most preserved cognitive and psychiatric outcomes, while RPDm patients were particularly impaired in these realms. Thus, the evolution of the disease in its early stages seems to differ between male and female patients according to motor symptom asymmetry, owing to physiological and cerebral differences between the two sexes. These findings point out to a growing body of literature addressing sex-based differences in the pathophysiology, treatment and clinical outcomes of patients with PD. Future studies will allow to better untangle the interactions occurring between biological processes in asymmetric Parkinson’s disease, and how they differentially impact male and female patients during the disease’s course.

## Conclusion

The present study helps provide a better understanding of the interactions occurring between UA, sex, and motor symptom asymmetry in the manifestation of cognitive and psychiatric symptoms in early PD. To our knowledge, it is the first study providing compelling evidence of different profiles in early PD based on motor symptom asymmetry at disease onset and sex, while accounting for the moderating role of serum UA. This may hold important clinical implications for NMS management in early-stage PD patients, with RPDm patients presenting greater vulnerability compared to their female counterparts.

## Supplementary Information

**Additional file 1: Table S1.1**. Cognitive scores for each PD subgroupand HC at each timepoint.** Table S1.2**. Intergroup comparisons at each timepoint of cognitivescores for each PD subgroupand HC.** Table S1.3**. Intragroup comparisonsof cognitive scores for each PD subgroupandHC.** Figure S1**. Evolution of cognitive scores for each PD subgroup andHC.** Table S2.1**. Psychiatricscores for each PD subgroupand HC at each timepoint.** Table S2.2**. Intergroup comparisons ateach timepoint of psychiatric scores for each PD subgroupand HC.** Table S2.3**. Intragroupcomparisonsof psychiatric scores for each PD subgroupand HC.** Figure S2**. Evolution of psychiatric scores for eachPD subgroup and HC.** Table S3.1**. Motor and serum uric acid scores for each PD subgroupand HC at each timepoint.** TableS3.2**. Intergroup comparisons at each timepoint of motor and serum uric acidoutcomes for each PD subgroupand HC.** Table S3.3**. Intragroup comparisonsof motor scores and serum uric acid for each PD subgroupand HC.** Figure S3**. Evolution of motor and serum uric acidoutcomes for each PD subgroup and HC.** Table S4**. Correlation between theEpworth sleepiness scale scores and the Symbol Digit Modalities Test scores forthe whole sample and each subgroup.

## Data Availability

All data were extracted from the PPMI funded by the Michael J. Fox Foundation for Parkinson’s Research and funding partners. Further details regarding data collection and measured outcomes have been previously published [44] and are available at http://www.ppmi-info.org/.
