# Supplementary material for: Moderating effects of uric acid and sex on cognition and psychiatric symptoms in asymmetric Parkinson’s disease
Source: Biol Sex Differ. 2023 May 4;14:26. doi: 10.1186/s13293-023-00510-1 (PMC10157998; doi:10.1186/s13293-023-00510-1)
Supplement: Supplementary file 1 — Additional file 1: Table S1.1. Cognitive scores for each PD subgroupand HC at each timepoint. Table S1.2. Intergroup comparisons at each timepoint of cognitivescores for each PD subgroupand HC. Table S1.3. Intragroup comparisonsof cognitive scores for each PD subgroupandHC. Figure S1. Evolution of cognitive scores for each PD subgroup andHC. Table S2.1. Psychiatricscores for each PD subgroupand HC at each timepoint. Table S2.2. Intergroup comparisons ateach timepoint of psychiatric scores for each PD subgroupand HC. Table S2.3. Intragroupcomparisonsof psychiatric scores for each PD subgroupand HC. Figure S2. Evolution of psychiatric scores for eachPD subgroup and HC. Table S3.1. Motor and serum uric acid scores for each PD subgroupand HC at each timepoint. TableS3.2. Intergroup comparisons at each timepoint of motor and serum uric acidoutcomes for each PD subgroupand HC. Table S3.3. Intragroup comparisonsof motor scores and serum uric acid for each PD subgroupand HC. Figure S3. Evolution of motor and serum uric acidoutcomes for each PD subgroup and HC. Table S4. Correlation between theEpworth sleepiness scale scores and the Symbol Digit Modalities Test scores forthe whole sample and each subgroup. [file 13293_2023_510_MOESM1_ESM.docx]

**Moderating effects of uric acid and sex on cognition and psychiatric symptoms in asymmetric Parkinson's disease**

Ioana Medeleine Constantin^a^, Philippe Voruz^a,b^, Julie Anne Péron^a,b*^

^1^ *Clinical and Experimental Neuropsychology Laboratory, Faculty of Psychology and Educational Sciences, University of Geneva, Geneva, Switzerland*

^2^ *Neurology Department, Geneva University Hospitals, Switzerland*

**Corresponding author*:

Professor Julie Anne Péron, Faculty of Psychology and Educational Sciences, University of Geneva. E-mail address: [julie.peron@unige.ch](mailto:julie.peron@unige.ch)

**ADDITIONAL FILE 1**

**Material S1**

**NMS according to motor symptom asymmetry and sex**

Inter- and intra-group comparisons were carried out in regard to motor symptom asymmetry and sex, for cognitive (Tables S1.1, S1.2, S1.3, and Figure S1), psychiatric (Tables S2.1, S2.2, S2.3, and Figure S2), as well as motor symptoms, serum UA and LEDD (Tables S3.1, S3.2, S3.3, and Figure S3).

**Data analysis**

Gaussian distribution was assessed using the Kolmogorov–Smirnov test. Nonparametric statistics were used to account for the between and within-subject effects given the non-normal distribution of an important number of continuous outcome variables. Kruskall-Wallis tests were used to assess differences between groups, followed by individual Man-Whitney U tests and chi-square tests. A priori comparisons were used to assess the difference between the following subgroups: LPDf vs LPDm ; RPDf vs RPDm ; LPDf vs RPDf ; LPDm vs RPDm ; LPDf vs HCf ; LPDm vs HCm ;RPDf vs HCf ; RPDm vs HCm. False discovery rate (FDR) corrections were used to correct for alpha-inflation in each test series. In addition, baseline and yearly follow-up assessments were used to assess the longitudinal evolution of outcome variables for each PD subgroup using non-parametric Wilcoxon analyses (*p* < .050).

**Inter-group comparisons of NMS in respect to motor symptom asymmetry and sex**

*Cognition* *(see Table S1.2 and* *Figure S1).* At baseline, male PD patients showed significantly reduced scores in comparison to female PD patients and HC on MoCA (*p* < .001), HVLT immediate recall (*p <* .008) and semantic fluency (*p* < .005). Both male and female PD patients had deficit performances on the SDMT (*p <* .008). Interestingly however, female PD patients (RPDf and LPDf) had significantly lower scores on the BJLOT (*p <* .006) in comparison to their male counterparts (RPDm and LPDm respectively). It is also noteworthy that male PD patients (RPDm and LPDm) as well as LPDf patients had significantly lower scores on the HVLT delayed recall (*p <* .008), whereas RPDf did not (*p* = .150). At Year 1, similar trends were observed, however RPDf patients had significantly higher scores on the MoCA compared to RPDm (*p* = .002), which was not the case for LPDf when compared to LPDm. Also, all PD patients exhibited deficits on the HVLT immediate recall (*p <* 0.020). Concerning HVLT delayed recall, male PD patients showed significantly lower scores when compared to their female counterparts (*p <* .005). Also, LPDf patients had lower scores on the semantic fluency total score (*p <* .01). At Year 3, male PD patients showed significantly reduced scores on MoCA (*p <* .020). RPDm patients demonstrated lower performances on the HVLT immediate and delayed recall compared to RPDf (*p <* .002) and HC (*p <* .007). This was not observed for the LPD subgroups. Both male and female PD patients had significantly lower performances on the SDMT (*p <* .010), with a significant difference noted for RPDf who performed better than RPDm (*p <* .001) ; such a difference was not noted for the LPD subgroups. Also, RPDm patients exhibited significantly lower performances on the semantic fluency task compared to HC (*p <* .030), and male PD patients scored significantly lower than their female counterparts (*p <* .003). Female PD patients continued to show lower scores on the BJLOT compared to male PD patients. At Year 5, the only statistically significant difference observed for the MOCA was between male and female RPD patients (*p* = .004), with male patients having worse performances. Regarding the HVLT immediate recall, RPDm and LPDf patients had significantly lower scores compared to HC (*p <* .010). For the HVLT delayed recall, LPDm and LPDf had significantly lower scores compared to HC (*p <* .008), whereas RPDm scored inferiorly to RPDf (*p* = .008). It is also noteworthy that solely LPDf patients continued to exhibit lower scores on the BJLOT when compared to LPDm (*p <* .008), the difference between RPDf and RPDm no longer being statistically significant (*p* = .187). All PD participants performed poorly on the symbol digit task as compared to HC (*p <* .006). However, RPDf had significantly superior scores when compared to RPDm (*p* = .002) ; once more, such difference was not noted between the LPD subgroups. Finally, male PD patients (LPDm and RPDm) were deficit in semantic fluency compared to their female counterparts (*p <* .001).

*Neuropsychiatry* *(see Table S2.2 and Figure S2).* At baseline, all PD patients showed significantly higher scores than HC on the GDS (*p <* .006) and STAI – total score inventories (*p <* .009). Of particular interest is the fact that on the STAI – trait subscale, LPDf, RPDm and LPDm patients had significantly higher scores when compared to HC (*p <* .01), but not RPDf. Also, RPDm, LPDm and LPDf patients had significantly higher scores on the RBD questionnaire (*p <* .001). This was not the case for RPDf patients however, who were significantly less affected then RPDm (*p* = .027). The same trends maintained through Year 1, however with the exception that the RPDf group no longer showed significantly higher scores on the STAI inventories (*p* = .065). All PD patients showed significantly higher scores on the RBD questionnaire when compared to HC (*p <* .015). By Year 3, similar trends were observed, with the RPDm, LPDm and LPDf groups scoring significantly higher on the STAI inventories (*p <* .007), which was not the case for the RPDf group. The RPD groups (male and female) had significantly higher scores on the Epworth Sleepiness Scale (*p <* .008), which wasn’t observed for the LPD groups. Reaching Year 5, the same trends were noticeable, with the exception of the GDS for which the RPDf group no longer showed significantly higher scores (*p* = .060), and the Epworth Sleepiness Scale where LPDm, in addition to RPDf and RPDm, had significantly higher scores (*p <* .002).

*Motor symptoms, medication and UA (see Table S3.2 and Figure S3).* All PD participants generally presented deteriorated symptomatology as measured by the MDS-UPDRS and Hoehn and Yahr scales. At baseline, male PD patients had significantly higher concentrations of serum UA compared to their female PD counterparts (*p <* .001). At Year 1, male patients still exhibited significantly higher UA concentrations compared to female PD patients (*p* < .001). Interestingly, RPDf exhibited significantly lower scores of UA compared to HC (*p* = .003), which was not the case for LPDf patients. By Year 3, similar trends were noticeable, with RPDm having significantly higher scores then RPDf on the second MDS-UPDRS scale measuring impairment in activities of daily life (*p* = .005); they also had a significantly higher intake of LEDD compared to RPDf (*p* = .011). Such differences were not observed between LPDm and LPDf patients. Male PD patients still exhibited significantly higher UA concentrations compared to female PD patients. It is noteworthy to mention that in comparison to RPDf, LPDf patients scored marginally higher on the fourth scale of the MDS-UPDRS, evaluating complications of therapy (*p* = .027; FDR corrected significance level at .025). By Year 5, differences between groups on the MDS-UPDRS scales became more important. The RPDm group exhibited higher scores compared to RPDf on the second (*p* < .001) and third (*p* = .004) MDS-UPDRS subscales, as well as on the total scale score (*p* < .002). Also, RPDm still exhibited higher LEDD compared to RPDf (*p* = .002). Once more, such differences were not noticeable between LPDm and LPDf patients. However, compared to RPDm, LPDm patients scored higher on the fourth scale of the MDS-UPDRS (*p* = .014). Male PD patients still had higher UA levels compared to female patients (*p* < .001).

**Intra-group comparisons of NMS in respect to motor symptom asymmetry and sex**

*Cognition* *(see Table S1.3 and Figure S1).* For RPDm, Wilcoxon analysis revealed a decrease over time for the following assessments: MoCA (Baseline vs Year 1, *p* < .001 and Baseline vs Year 3, *p* < .001), BJLOT (Baseline vs Year 5, *p* = .003), SDMT (Baseline vs Year 5, *p* = .004) and semantic fluency total score (Baseline vs Year 5, *p* = .021). For LPDm, longitudinal analyses revealed a decline on the following measured outcomes: MoCA (Baseline vs Year 1, *p* = .007 and Baseline vs Year 3, *p* = .009), BJLOT (Baseline vs Year 5, *p* = .012) and SDMT (Baseline vs Year 3, *p* = .006 and Baseline vs Year 5, *p* = .014). Regarding the RPDf group, a single decline was noted on the BJLOT solely at the beginning of the follow-up (Baseline vs Year 1, *p* = .007). The same decline was observed for the LPDf group, however this persisted on the longer run (Baseline vs Year 1, *p* = .003 and Baseline vs Year 5, *p* = .003).

*Neuropsychiatry (see Table S2.3 and Figure S2)*. For RPDm, a significant increase was noted on the Epworth Sleepiness Scale (Baseline vs Year 3, *p* < .001 and Baseline vs Year 5, *p* < .001), the GDS (Baseline vs Year 5, *p* = .004), and RBD questionnaire (Baseline vs Year 5, *p* < .001). Regarding the LPDm group, a significant increase in scores was observed solely for the Epworth Sleepiness Scale (Baseline vs Year 1, *p* = .001, Baseline vs Year 3, *p* < .001 and Baseline vs Year 5, *p* < .001). The RPDf group did not exhibit any increase over time; rather, the group showed a decrease of scores on the STAI inventory (Trait subscale, Baseline vs Year 3, *p* = .008; Total score Baseline vs Year 3, *p* = .008). The LPDf group however showed an increase over time on the Epworth Sleepiness Scale (Baseline vs Year 3, *p* < .001).

*Motor symptoms, medication and UA (see Table S3.3 and Figure S3).* Multiple changes over time were noticed for all PD patients which translated into a worsening of symptomatology, as measured by the MDS-UPDRS, as well as a significant increase in medication requirements. However, it is noteworthy that at Year 1 and Year 3 male PD patients showed significant worsening on the second (Baseline vs Year 1, *p* < .001) and third (on period; Baseline vs Year 3, *p* < .004) scales of the MDS-UPDRS respectively; such was not the case for female PD patients. By Year 5, the RPDm group still presented significant worsening scores on the third MDS-UPDRS subscale (on period; Baseline vs Year 5, *p* < .001), which was no longer the case for LPDm.

**Table S1.1.** Cognitive scores for each PD subgroup (LPDf ; LPDm ; RPDf ; RPDm) and HC at each timepoint.

|  | Baseline | | | | | | Year 1 | | | | | |
| --- | --- | --- | --- | --- | --- | --- | --- | --- | --- | --- | --- | --- |
|  | LPDf  (*n* = 74) | LPDm  (*n* = 105) | RPDf  (*n* = 71) | RPDm  (*n* = 163) | HCf  (*n* = 70) | HCm  (*n* = 126) | LPDf  (*n* = 70) | LPDm  (*n* = 95) | RPDf  (*n* = 66) | RPDm  (*n* = 156) | HCf  (*n* = 67) | HCm  (*n* = 118) |
| MoCA (mean ± SD) | 27.50 (± 2.35) | 26.87 (± 2.42) | 27.56 (± 2.03) | 26.88 (± 2.34) | 28.34 (± 1.08) | 28.17 (± 1.12) | 26.99 (± 2.85) | 26.06 (± 2.83) | 27.12 (± 2.43) | 25.85 (± 2.85) | 27.51 (± 2.15) | 27.14 (± 2.21) |
| HVLT – Immediate/Total recall (mean ± SD) | 25.58 (± 4.77) | 23.21 (± 4.70) | 26.67 (± 4.58) | 23.74 (± 5.11) | 27.26 (± 4.37) | 25.37 (± 4.44) | 24.97 (± 5.34) | 22.97 (± 5.23) | 25.06 (± 5.51) | 23.34 (± 5.37) | 27.52 (± 4.46) | 25.67 (± 4.66) |
| HVLT – Delayed recall (mean ± SD) | 8.95 (± 2.26) | 7.68 (± 2.58) | 9.33 (± 2.22) | 8.07 (± 2.58) | 9.89 (± 1.92) | 8.96 (± 2.46) | 8.74 (± 2.82) | 7.36 (± 2.69) | 8.97 (± 2.91) | 7.88 (± 2.86) | 9.67 (± 2.23) | 8.81 (± 2.60) |
| Benton Judgement of Line Orientation (mean ± SD) | 12.22 (± 2.26) | 13.11 (± 1.91) | 11.97 (± 2.09) | 13.10 (± 2.11) | 12.43 (± 2.18) | 13.51 (± 1.76) | 11.6 (± 2.84) | 12.84 (± 1.96) | 11.3 (± 2.49) | 12.79 (± 2.23) | 11.63 (± 2.59) | 13.23 (± 2.18) |
| Symbol Digit Modalities Test (mean ± SD) | 43.68 (± 10.91) | 41.08 (± 9.87) | 42.86 (± 7.75) | 39.50 (± 9.53) | 48.84 (± 10.09) | 45.62 (± 10.62) | 43.06 (± 10.53) | 40.43 (± 9.64) | 42.30 (± 9.51) | 39.77 (± 10.57) | 49.78 (± 10.01) | 46.30 (± 11.36) |
| Semantic Fluency – Total score (mean ± SD) | 52.84 (± 11.34) | 45.23 (± 10.82) | 53.53 (± 12.00) | 47.06 (± 11.14) | 56.84 (± 10.69) | 48.99 (± 10.51) | 52.87 (± 11.41) | 45.92 (± 12.15) | 53.24 (± 9.95) | 47.00 (± 10.77) | 57.04 (± 9.72) | 49.89 (± 11.22) |
|  | Year 3 | | | | | | Year 5 | | | | | |
|  | LPDf  (*n* = 67) | LPDm  (*n* = 87) | RPDf  (*n* = 59) | RPDm  (*n* = 144) | HCf  (*n* = 64) | HCm  (*n* = 103) | LPDf  (*n* = 59) | LPDm  (*n* = 75) | RPDf  (*n* = 46) | RPDm  (*n* = 129) | HCf  (*n* = 58) | HCm  (*n* = 96) |
| MoCA (mean ± SD) | 27.09 (± 3.33) | 26.23 (± 2.91) | 27.11 (± 2.90) | 25.91 (± 2.91) | 27.75 (± 2.05) | 27.26 (± 2.25) | 26.83 (± 3.51) | 26.47 (± 3.18) | 27.89 (± 2.10) | 26.10 (± 4.01) | 28.03 (± 2.06) | 27.33 (± 2.24) |
| HVLT – Immediate/Total recall (mean ± SD) | 26.29 (± 5.95) | 24.64 (± 5.25) | 26.62 (± 6.32) | 23.20 (± 6.23) | 27.98 (± 4.79) | 25.57 (± 5.37) | 25.00 (± 6.18) | 24.09 (± 5.95) | 27.61 (± 4.96) | 23.43 (± 7.04) | 29.40 (± 3.80) | 25.92 (± 5.89) |
| HVLT – Delayed recall (mean ± SD) | 9.12 (± 2.51) | 8.05 (± 2.97) | 9.16 (± 3.06) | 7.78 (± 3.21) | 9.70 (± 2.19) | 8.90 (± 2.67) | 8.97 (± 2.85) | 8.01 (± 3.24) | 9.74 (± 2.98) | 8.25 (± 3.30) | 10.41 (± 2.18) | 9.20 (± 2.90) |
| Benton Judgement of Line Orientation (mean ± SD) | 11.86 (± 2.49) | 13.02 (± 1.95) | 11.89 (± 2.38) | 13.00 (± 2.10) | 11.70 (± 2.41) | 13.07 (± 2.03) | 11.60 (± 2.38) | 12.61 (± 2.32) | 12.33 (± 2.22) | 12.71 (± 2.20) | 11.97 (± 2.62) | 13.22 (± 1.86) |
| Symbol Digit Modalities Test (mean ± SD) | 42.94 (± 12.06) | 39.08 (± 11.03) | 44.02 (± 10.47) | 38.04 (± 11.65) | 49.78 (± 9.56) | 46.77 (± 11.7) | 43.45 (± 13.08) | 39.07 (± 12.72) | 44.39 (± 10.47) | 37.52 (± 12.30) | 50.26 (± 10.21) | 45.99 (± 12.20) |
| Semantic Fluency – Total score (mean ± SD) | 52.30 (± 12.36) | 46.31 (± 11.24) | 52.90 (± 12.44) | 46.00 (± 10.85) | 57.17 (± 10.80) | 50.14 (± 11.83) | 54.03 (± 12.73) | 44.92 (± 13.05) | 56.39 (± 10.97) | 45.59 (± 12.47) | 58.02 (± 11.36) | 49.80 (± 12.14) |

***Legend*.** f: female; HC: healthy controls; LPD: patients with Parkinson’s disease (PD) who exhibit predominantly left-sided motor symptoms; m: male; MoCA: Montreal Cognitive Assessment; MCI: Mild Cognitive Impairment; HVLT: Hopkins Verbal Learning Test; RPD: patients with PD who exhibit predominantly right-sided motor symptoms; SD: standard deviation.

**Table S1.2.** Intergroup comparisons at each timepoint of cognitive scores for each PD subgroup (LPDf ; LPDm ; RPDf ; RPDm) and HC.

|  | Baseline  K-W and M-W/Khi2 Bonferroni correction | | | | | | | | | Year 1  K-W and M-W/Khi2 Bonferroni correction | | | | | | | | |
| --- | --- | --- | --- | --- | --- | --- | --- | --- | --- | --- | --- | --- | --- | --- | --- | --- | --- | --- |
|  | K-W/ Khi^2^ | LPDf vs LPDm | RPDf vs RPDm | LPDf vs RPDf | LPDm vs RPDm | LPDf vs HCf | LPDm vs HCm | RPDf vs HCf | RPDm vs HCm | K-W/ Khi^2^ | LPDf vs LPDm | RPDf vs RPDm | LPDf vs RPDf | LPDm vs RPDm | LPDf vs HCf | LPDm vs HCm | RPDf vs HCf | RPDm vs HCm |
| MoCA | < .001^*^ | .048^*^ | .032^*^ | .931 | .978 | .093 | <.001^**^ | .077 | <.001^**^ | <.001^*^ | .012^*^ | .002^**^ | .929 | .479 | .484 | .006^**^ | .404 | <.001^**^ |
| HVLT – Immediate/Total recall | < .001^*^ | .001^**^ | <.001^**^ | .164 | .307 | .045^*^ | .001^**^ | .528 | .008^**^ | <.001^*^ | .023^*^ | .010^*^ | .614 | .583 | .004^**^ | <.001^**^ | .017^*^ | <.001^**^ |
| HVLT – Delayed recall | < .001^*^ | .001^**^ | <.001^**^ | .284 | .247 | .008^**^ | <.001^**^ | .149 | .002^**^ | < .001^*^ | .001^**^ | .005^**^ | .522 | .149 | .058 | <.001^**^ | .263 | .010^**^ |
| Benton Judgement of Line Orientation | < .001^*^ | .006^**^ | <.001^**^ | .352 | .796 | .607 | .094 | .106 | .105 | <.001^*^ | .006^**^ | <.001^**^ | .362 | .707 | .903 | .020^*^ | .320 | .033^*^ |
| Symbol Digit Modalities Test | < .001^*^ | .113 | .012^*^ | .453 | .084 | .008^**^ | .005^**^ | .001^**^ | <.001^**^ | < .001^*^ | .070 | .079 | .616 | .648 | .001^**^ | <.001^**^ | <.001^**^ | <.001^**^ |
| Semantic Fluency – Total score | < .001^*^ | <.001^**^ | <.001^**^ | .820 | .131 | .022^*^ | .005^**^ | .033^*^ | .139 | < .001^*^ | <.001^**^ | <.001^**^ | .495 | .334 | .010^**^ | .011^*^ | .052 | .050^*^ |
|  | Year 3  K-W and M-W/Khi2 Bonferroni correction | | | | | | | | | Year 5  K-W and M-W/Khi2 Bonferroni correction | | | | | | | | |
|  | K-W/ Khi^2^ | LPDf vs LPDm | RPDf vs RPDm | LPDf vs RPDf | LPDm vs RPDm | LPDf vs HCf | LPDm vs HCm | RPDf vs HCf | RPDm vs HCm | K-W/ Khi^2^ | LPDf vs LPDm | RPDf vs RPDm | LPDf vs RPDf | LPDm vs RPDm | LPDf vs HCf | LPDm vs HCm | RPDf vs HCf | RPDm vs HCm |
| MoCA | < .001^*^ | .008^**^ | .002^**^ | .554 | .326 | .568 | .012^**^ | .280 | <.001^**^ | .001^*^ | .260 | .004^**^ | .218 | .817 | .098 | .139 | .723 | .054 |
| HVLT – Immediate/Total recall | < .001^*^ | .057 | <.001^**^ | .657 | .072 | .098 | .356 | .267 | .004^**^ | < .001^*^ | .299 | .001^**^ | .023^*^ | .599 | <.001^**^ | .046^*^ | .081 | .010^**^ |
| HVLT – Delayed recall | < .001^*^ | .023^*^ | .002^**^ | .528 | .679 | .251 | .045^*^ | .734 | .007^**^ | < .001^*^ | .080 | .003^**^ | .048^*^ | .514 | .001^**^ | .008^**^ | .426 | .026^*^ |
| Benton Judgement of Line Orientation | < .001^*^ | .002^**^ | .001^**^ | .945 | .786 | .638 | .673 | .598 | .876 | < .001^*^ | .008^**^ | .187 | .100 | .895 | .328 | .099 | .619 | .075 |
| Symbol Digit Modalities Test | < .001^*^ | .029^*^ | .001^**^ | .627 | .526 | .002^**^ | <.001^**^ | .010^**^ | <.001^**^ | < .001^*^ | .074 | .002^**^ | .699 | .386 | .003^**^ | .001^**^ | .006^**^ | <.001^**^ |
| Semantic Fluency – Total score | < .001^*^ | .003^**^ | < .001^**^ | .681 | .665 | .023^*^ | .045^*^ | .026^*^ | .007^**^ | < .001^*^ | <.001^**^ | <.001^**^ | .283 | .548 | .061 | .012^**^ | .413 | .027^*^ |

***Legend*.** f: female; HC: healthy controls; LPD: patients with Parkinson’s disease (PD) who exhibit predominantly left-sided motor symptoms; m: male; MoCA: Montreal Cognitive Assessment; MCI: Mild Cognitive Impairment; HVLT: Hopkins Verbal Learning Test; RPD: patients with PD who exhibit predominantly right-sided motor symptoms; K-W: Kruskal-Wallis test; M-W: Mann–Whitney U test.

^*^ *p* <.05; ^**^ *p* <.05 FDR corrected

**Table S1.3.** Intragroup comparisons (Year 1 compared to Baseline; Year 3 compared to baseline; Year 5 compared to baseline) of cognitive scores for each PD subgroup (LPDf ; LPDm ; RPDf ; RPDm) and HC.

|  | Comparisons  Year 1 and Baseline | | | | | | Comparisons  Year 3 and Baseline | | | | | | Comparisons  Year 5 and Baseline | | | | | |
| --- | --- | --- | --- | --- | --- | --- | --- | --- | --- | --- | --- | --- | --- | --- | --- | --- | --- | --- |
|  | LPDf  (*n* = 74) | LPDm  (*n* = 105) | RPDf  (*n* = 71) | RPDm  (*n* = 163) | HCf  (*n* = 70) | HCm  (*n* = 126) | LPDf  (*n* = 74) | LPDm  (*n* = 105) | RPDf  (*n* = 71) | RPDm  (*n* = 163) | HCf  (*n* = 70) | HCm  (*n* = 126) | LPDf  (*n* = 74) | LPDm  (*n* = 105) | RPDf  (*n* = 71) | RPDm  (*n* = 163) | HCf  (*n* = 70) | HCm  (*n* = 126) |
| MoCA | .236 | .007^**^ | .187 | <.001^**^ | .007^**^ | <.001^**^ | .815 | .009^**^ | .382 | <.001^**^ | .018^*^ | <.001^**^ | .255 | .121 | .457 | .125 | .323 | <.001^**^ |
| HVLT – Immediate/Total recall | .355 | .473 | .037 | .102 | .629 | .500 | .102 | .042 | .958 | .265 | .077 | .626 | .807 | .783 | .706 | .462 | .008^**^ | .433 |
| HVLT – Delayed recall | .882 | .199 | .295 | .368 | .432 | .780 | .669 | .311 | .382 | .314 | .527 | .936 | .609 | .448 | .980 | .431 | .111 | .114 |
| Benton Judgement of Line Orientation | .003^**^ | .109 | .007^**^ | .041^*^ | .004^**^ | .282 | .118 | .344 | .129 | .191 | .031^*^ | .017^**^ | .003^**^ | .012^**^ | .574 | .003^**^ | .174 | .013^**^ |
| Symbol Digit Modalities Test | .904 | .357 | .886 | .591 | .247 | .301 | .704 | .006^**^ | .296 | .036^*^ | .076 | .081 | .147 | .014^**^ | .563 | .004^**^ | .608 | .372 |
| Semantic Fluency – Total score | .851 | .789 | .749 | .727 | .875 | .030^**^ | .455 | .769 | .997 | .057 | .810 | .552 | .712 | .205 | .129 | .021^**^ | .944 | .503 |

***Legend*.** f: female; HC: healthy controls; LPD: patients with Parkinson’s disease (PD) who exhibit predominantly left-sided motor symptoms; m: male; MoCA: Montreal Cognitive Assessment; HVLT: Hopkins Verbal Learning Test; RPD: patients with PD who exhibit predominantly right-sided motor symptoms.

^*^ *p* <.05; ^**^ *p* <.05 FDR corrected

**B.**

**A.**


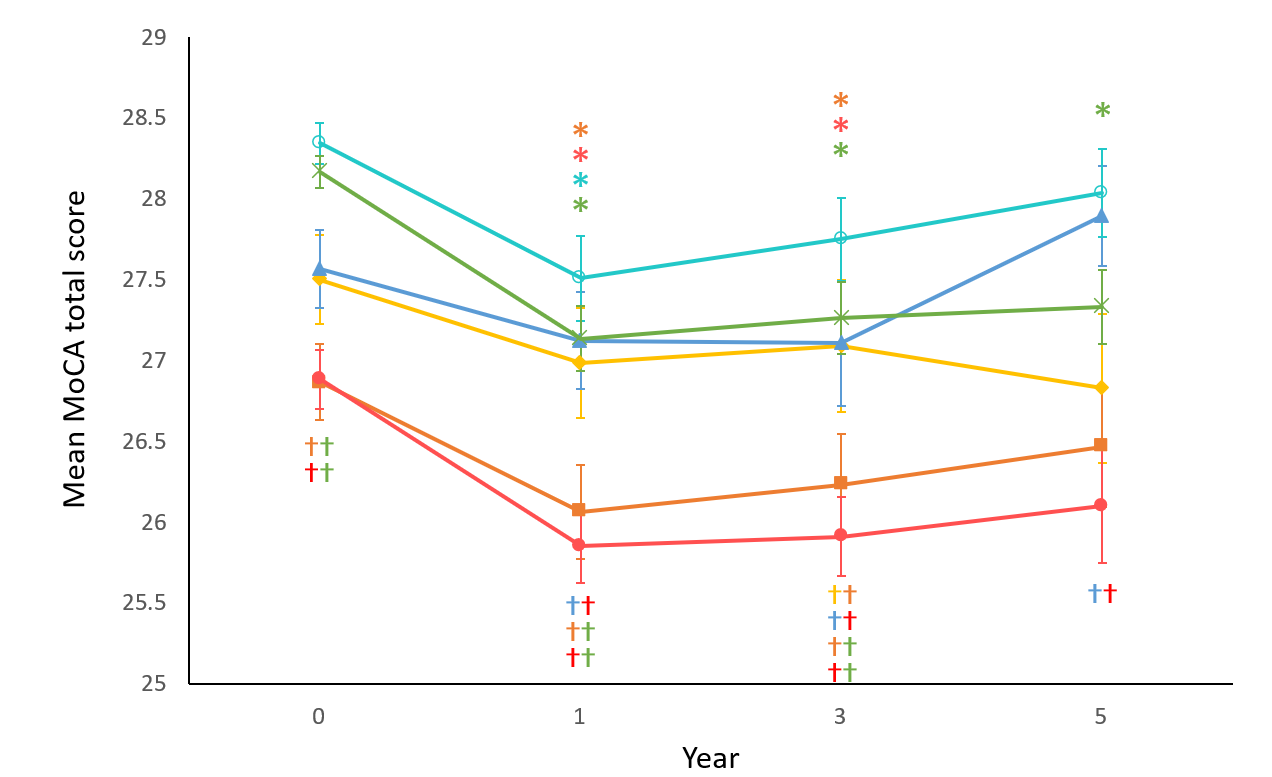

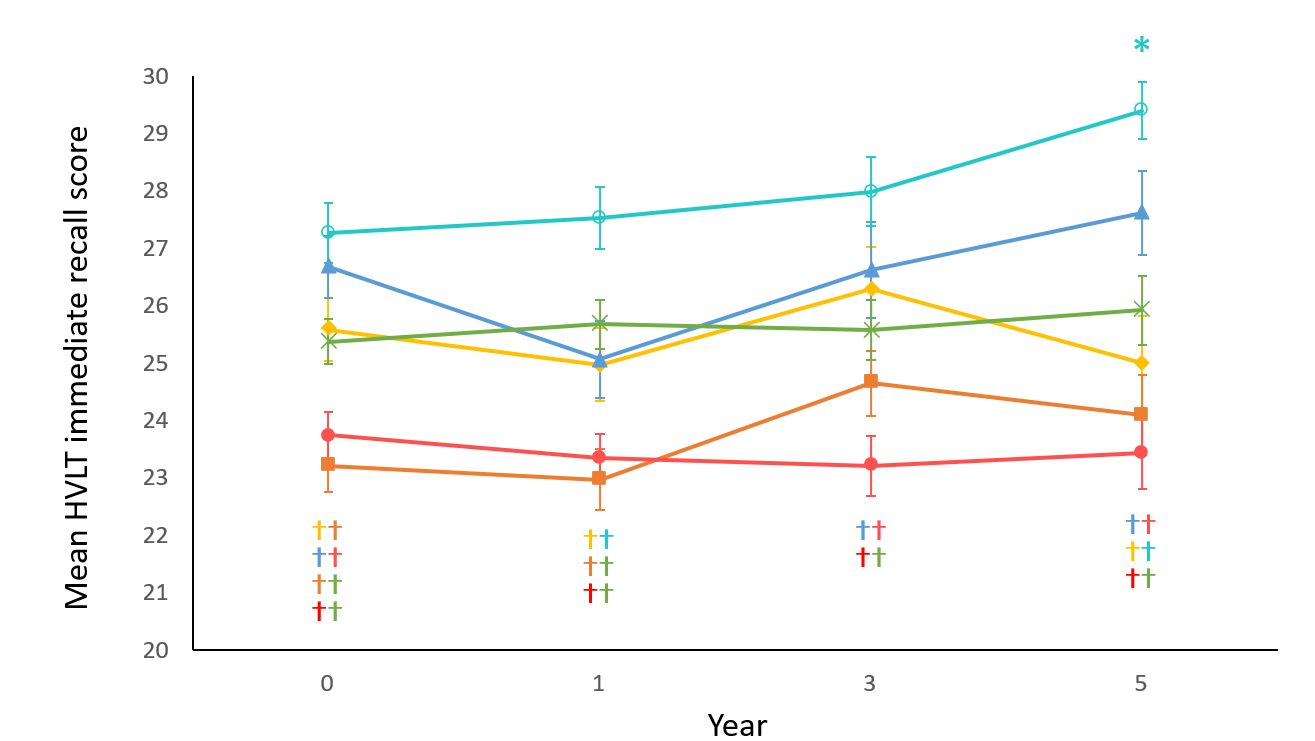


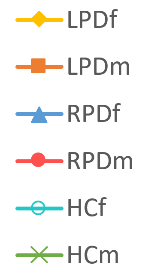


**D.**

**C.**


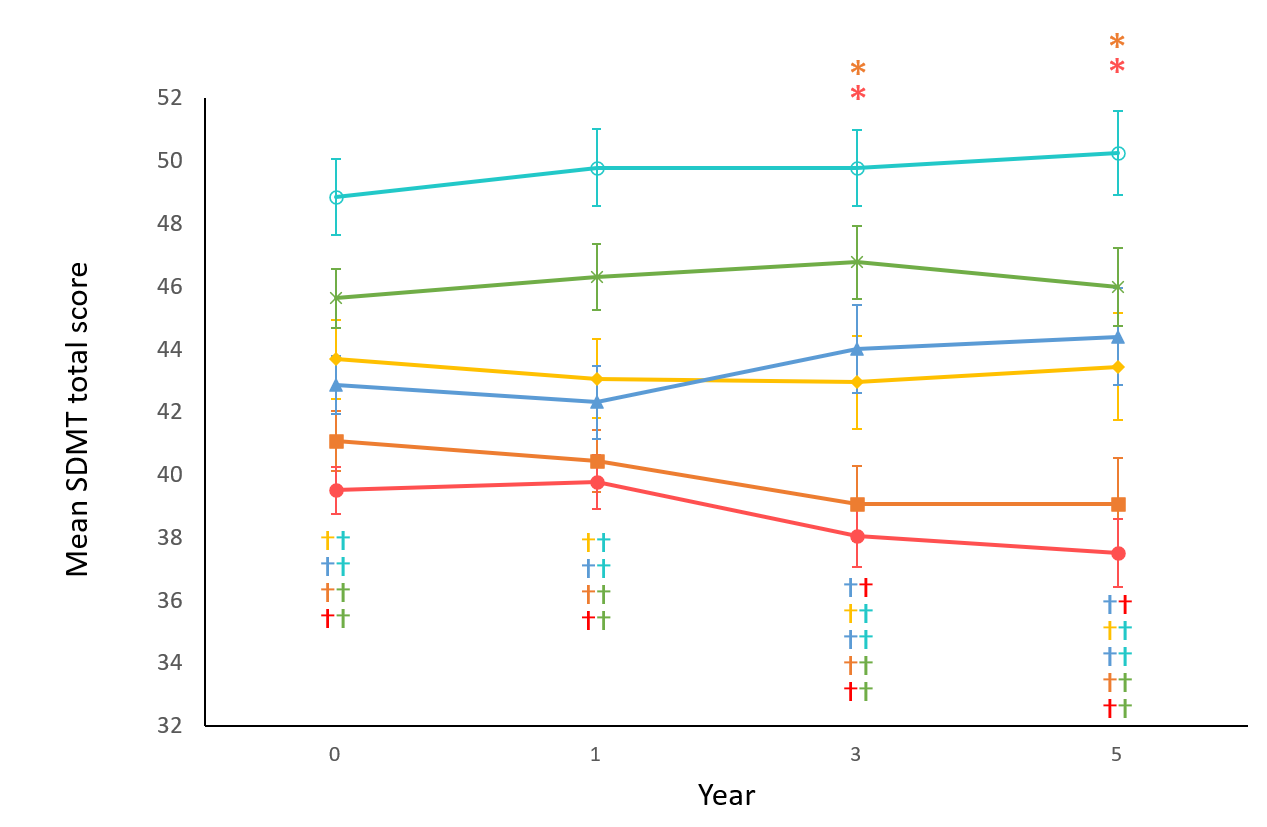

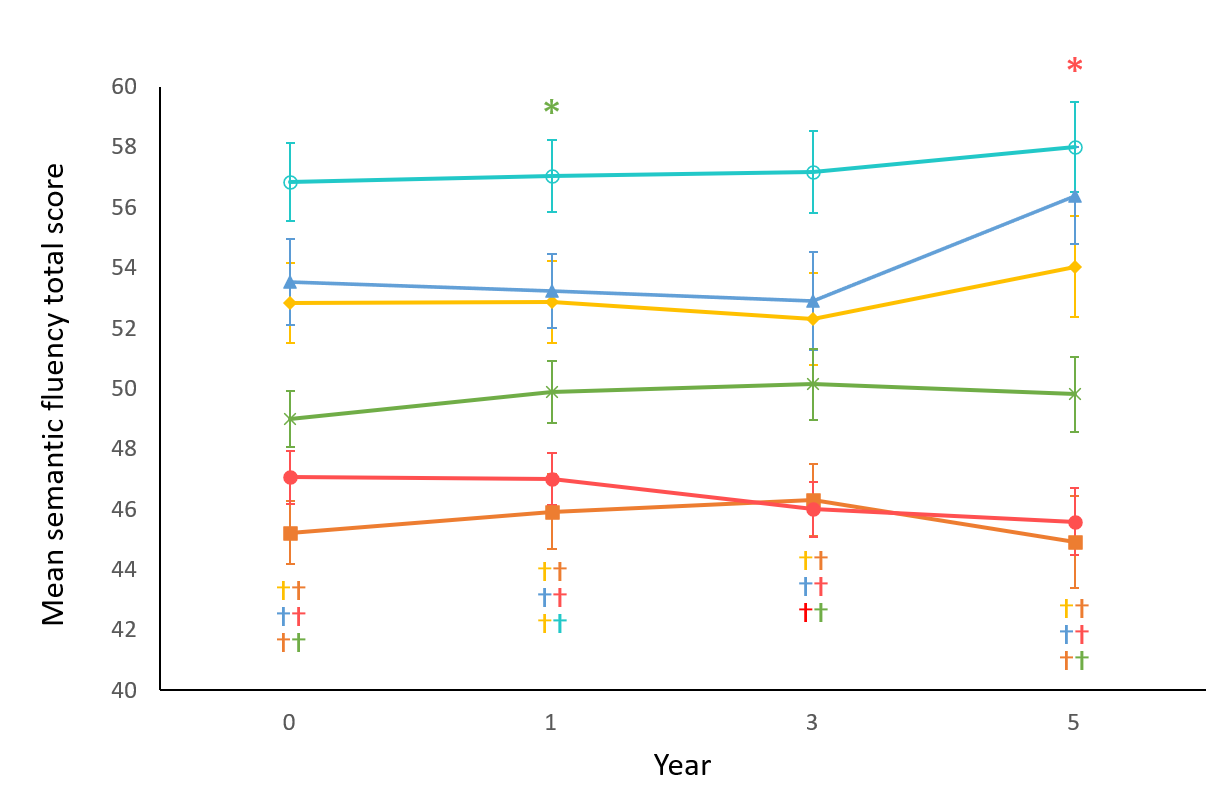
**Figure S1. Evolution of cognitive scores for each PD subgroup and HC**. f: female; HC: healthy controls; LPD: patients with Parkinson’s disease who exhibit predominantly left-sided motor symptoms; m: male; MoCA: Montreal Cognitive Assessment; HVLT: Hopkins Verbal Learning Test; RPD: patients with PD who exhibit predominantly right-sided motor symptoms; SDMT: Symbol Digit Modalities Test. Error bars are ± 1 SEM. * indicates a significant difference (*p* < .05) compared with baseline values. ✝✝ indicate significant differences (*p* < .05) between two subgroups at a given timepoint.

**Table S2.1.** Psychiatric scores for each PD subgroup (LPDf ; LPDm ; RPDf ; RPDm) and HC at each timepoint.

|  | Baseline | | | | | | Year 1 | | | | | |
| --- | --- | --- | --- | --- | --- | --- | --- | --- | --- | --- | --- | --- |
|  | LPDf  (*n* = 74) | LPDm  (*n* = 105) | RPDf  (*n* = 71) | RPDm  (*n* = 163) | HCf  (*n* = 70) | HCm  (*n* = 126) | LPDf  (*n* = 70) | LPDm  (*n* = 95) | RPDf  (*n* = 66) | RPDm  (*n* = 156) | HCf  (*n* = 67) | HCm  (*n* = 118) |
| GDS (mean ± SD) | 2.49 (± 2.55) | 2.50 (± 2.54) | 2.37 (± 2.85) | 2.15 (± 2.17) | 1.47 (± 2.38) | 1.19 (± 1.93) | 2.89 (± 3.24) | 2.56 (± 2.89) | 2.68 (± 3.36) | 2.44 (± 2.65) | 1.48 (± 2.36) | 1.37 (± 2.37) |
| STAI – State (mean ± SD) | 34.90 (± 11.04) | 33.66 (± 10.45) | 32.77 (± 11.03) | 31.85 (± 9.43) | 28.19 (± 8.54) | 27.92 (± 7.77) | 35.10 (± 11.5) | 32.71 (± 10.15) | 32.17 (± 11.00) | 31.44 (± 8.73) | 29.40 (± 9.34) | 26.31 (± 7.96) |
| STAI – Trait (mean ± SD) | 34.68 (± 10.34) | 32.21 (± 9.74) | 33.23 (± 10.68) | 31.15 (± 8.21) | 30.14 (± 8.90) | 28.54 (± 6.51) | 34.57 (± 11.18) | 32.8 (± 8.95) | 32.79 (± 10.82) | 31.91 (± 8.93) | 30.52 (± 9.86) | 27.79 (± 7.96) |
| STAI – Total score (mean ± SD) | 69.59 (± 20.08) | 65.87 (± 18.96) | 66.00 (± 19.73) | 63.00 (± 16.38) | 58.33 (± 15.86) | 56.46 (± 13.01) | 69.67 (± 21.64) | 65.51 (± 18.28) | 64.95 (± 20.10) | 63.35 (± 16.80) | 59.93 (± 18.51) | 54.09 (± 15.30) |
| Epworth Scale (mean ± SD) | 5.16 (± 3.29) | 5.58 (± 3.24) | 5.92 (± 3.91) | 6.04 (± 3.46) | 5.17 (± 3.05) | 5.87 (± 3.61) | 5.33 (± 4.04) | 6.58 (± 4.10) | 5.67 (± 3.82) | 6.26 (± 3.91) | 5.16 (± 3.26) | 5.55 (± 3.22) |
| REM Questionnaire (mean ± SD) | 4.04 (± 2.38) | 4.07 (± 2.80) | 3.54 (± 2.49) | 4.47 (± 2.83) | 2.74 (± 2.33) | 2.87 (± 2.23) | 4.24 (± 2.83) | 4.24 (± 2.60) | 3.71 (± 2.62) | 4.13 (± 2.96) | 2.58 (± 1.96) | 2.94 (± 2.42) |
|  | Year 3 | | | | | | Year 5 | | | | | |
|  | LPDf  (*n* = 67) | LPDm  (*n* = 87) | RPDf  (*n* = 59) | RPDm  (*n* = 144) | HCf  (*n* = 64) | HCm  (*n* = 103) | LPDf  (*n* = 59) | LPDm  (*n* = 75) | RPDf  (*n* = 46) | RPDm  (*n* = 129) | HCf  (*n* = 58) | HCm  (*n* = 96) |
| GDS (mean ± SD) | 3.21 (± 3.72) | 2.79 (± 2.94) | 2.08 (± 2.27) | 2.49 (± 2.45) | 1.23 (± 1.97) | 0.89 (± 1.15) | 3.16 (± 3.14) | 2.69 (± 2.40) | 2.28 (± 2.96) | 2.84 (± 2.86) | 1.17 (± 1.69) | 1.00 (± 1.41) |
| STAI – State (mean ± SD) | 35.18 (± 12.89) | 32.53 (± 9.99) | 30.47 (± 9.44) | 30.97 (± 8.29) | 28.52 (± 8.45) | 26.16 (± 6.3) | 32.98 (± 12.04) | 31.88 (± 9.54) | 30.17 (± 9.18) | 32.26 (± 9.68) | 27.31 (± 8.31) | 26.67 (± 6.89) |
| STAI – Trait (mean ± SD) | 34.99 (± 11.10) | 32.64 (± 9.58) | 31.62 (± 10.70) | 32.09 (± 8.85) | 29.64 (± 8.14) | 27.44 (± 6.17) | 34.29 (± 11.59) | 32.24 (± 9.29) | 31.48 (± 11.90) | 32.57 (± 9.61) | 29.07 (± 8.64) | 26.49 (± 6.81) |
| STAI – Total score (mean ± SD) | 70.16 (± 22.48) | 65.17 (± 18.6) | 61.83 (± 18.78) | 63.06 (± 16.38) | 58.16 (± 15.76) | 53.64 (± 11.66) | 67.28 (± 22.53) | 64.14 (± 18.19) | 61.65 (± 20.13) | 64.83 (± 18.41) | 56.38 (± 15.98) | 53.16 (± 12.96) |
| Epworth Scale (mean ± SD) | 7.22 (± 5.43) | 7.32 (± 4.01) | 6.72 (± 4.34) | 7.58 (± 4.41) | 4.67 (± 3.16) | 6.11 (± 3.78) | 6.54 (± 4.66) | 8.33 (± 3.96) | 7.48 (± 5.28) | 8.07 (± 4.91) | 4.48 (± 3.23) | 5.78 (± 4.02) |
| REM Questionnaire (mean ± SD) | 4.44 (± 2.77) | 4.55 (± 2.97) | 4.02 (± 2.87) | 4.81 (± 3.04) | 2.05 (± 1.72) | 2.95 (± 2.50) | 4.39 (± 2.96) | 4.59 (± 3.27) | 4.26 (± 2.94) | 5.32 (± 3.20) | 2.10 (± 2.01) | 2.83 (± 2.42) |

***Legend*.** f: female; HC: healthy controls; GDS: Geriatric Depression Scale; LPD: patients with Parkinson’s disease (PD) who exhibit predominantly left-sided motor symptoms; m: male; RBD: REM Sleep Behavior Disorder; RPD: patients with PD who exhibit predominantly right-sided motor symptoms; SD: standard deviation; STAI: State-Trait Anxiety Inventory.

**Table S2.2.** Intergroup comparisons at each timepoint of psychiatric scores for each PD subgroup (LPDf ; LPDm ; RPDf ; RPDm) and HC.

|  | Baseline  K-W and M-W/Khi2 Bonferroni correction | | | | | | | | | Year 1  K-W and M-W/Khi2 Bonferroni correction | | | | | | | | |
| --- | --- | --- | --- | --- | --- | --- | --- | --- | --- | --- | --- | --- | --- | --- | --- | --- | --- | --- |
|  | K-W/ Khi^2^ | LPDf vs LPDm | RPDf vs RPDm | LPDf vs RPDf | LPDm vs RPDm | LPDf vs HCf | LPDm vs HCm | RPDf vs HCf | RPDm vs HCm | K-W/ Khi^2^ | LPDf vs LPDm | RPDf vs RPDm | LPDf vs RPDf | LPDm vs RPDm | LPDf vs HCf | LPDm vs HCm | RPDf vs HCf | RPDm vs HCm |
| GDS | < .001^*^ | .799 | .772 | .544 | .365 | .001^**^ | < 001^**^ | .006^**^ | < 001^**^ | < .001^*^ | .690 | .432 | .472 | .745 | .001^**^ | < 001^**^ | .011^**^ | <.001^**^ |
| STAI – State | < .001^*^ | .511 | .759 | .217 | .187 | <.001^**^ | <.001^**^ | .004^**^ | <.001^**^ | < .001^*^ | .227 | .861 | .091 | .419 | .001^**^ | <.001^**^ | .062 | <.001^**^ |
| STAI – Trait | < .001^*^ | .093 | .328 | .255 | .688 | .004^**^ | .010^**^ | .071 | .008^**^ | < .001^*^ | .501 | .926 | .244 | .404 | .015^**^ | <.001^**^ | .185 | <.001^**^ |
| STAI – Total score | < .001^*^ | .238 | .432 | .225 | .339 | <.001^**^ | <.001^**^ | .009^**^ | .001^**^ | < .001^*^ | .276 | .937 | .185 | .370 | .003^**^ | <.001^**^ | .065 | <.001^**^ |
| Epworth Sleepiness Scale | .368 | - | - | - | - | - | - | - | - | .102 | - | - | - | - | - | - | - | - |
| RBD Questionnaire | < .001^*^ | .694 | .027^*^ | .133 | .239 | .001^**^ | .001^**^ | .035^*^ | <.001^**^ | < .001^*^ | .835 | .456 | .270 | .400 | <.001^**^ | <.001^**^ | .015^*^ | .001^**^ |
|  | Year 3  K-W and M-W/Khi2 Bonferroni correction | | | | | | | | | Year 5  K-W and M-W/Khi2 Bonferroni correction | | | | | | | | |
|  | K-W/ Khi^2^ | LPDf vs LPDm | RPDf vs RPDm | LPDf vs RPDf | LPDm vs RPDm | LPDf vs HCf | LPDm vs HCm | RPDf vs HCf | RPDm vs HCm | K-W/ Khi^2^ | LPDf vs LPDm | RPDf vs RPDm | LPDf vs RPDf | LPDm vs RPDm | LPDf vs HCf | LPDm vs HCm | RPDf vs HCf | RPDm vs HCm |
| GDS | < .001^*^ | .982 | .264 | .250 | .671 | <.001^**^ | <.001^**^ | .003^**^ | <.001^**^ | < .001^*^ | .737 | .044^**^ | .063 | .965 | <.001^**^ | <.001^**^ | .060 | <.001^**^ |
| STAI – State | < .001^*^ | .358 | .375 | .047^*^ | .381 | .002^**^ | <.001^**^ | .257 | <.001^**^ | < .001^*^ | .973 | .160 | .309 | .784 | .007^**^ | <.001^**^ | .065 | <.001^**^ |
| STAI – Trait | < .001^*^ | .234 | .284 | .034^*^ | .809 | .007^**^ | <.001^**^ | .538 | <.001^**^ | < .001^*^ | .458 | .113 | .121 | .852 | .010^**^ | <.001^**^ | .380 | <.001^**^ |
| STAI – Total score | < .001^*^ | .235 | .277 | .021^*^ | .527 | .001^**^ | <.001^**^ | .320 | <.001^**^ | < .001^*^ | .604 | .112 | .182 | .735 | .005^**^ | <.001^**^ | .134 | <.001^**^ |
| Epworth Sleepiness Scale | < .001^*^ | .283 | .267 | .917 | .917 | .014^*^ | .020^*^ | .006^**^ | .008^**^ | < .001^*^ | .004^**^ | .362 | .358 | .356 | .016^*^ | <.001^**^ | .002^**^ | <.001^**^ |
| RBD Questionnaire | < .001^*^ | .928 | .070 | .309 | .517 | <.001^**^ | <.001^**^ | <.001^**^ | <.001^**^ | < .001^*^ | .828 | .058 | .738 | .105 | <.001^**^ | <.001^**^ | <.001^**^ | <.001^**^ |

***Legend*.** f: female; HC: healthy controls; GDS: Geriatric Depression Scale; LPD: patients with Parkinson’s disease (PD) who exhibit predominantly left-sided motor symptoms; m: male; RBD: REM Sleep Behavior Disorder; RPD: patients with PD who exhibit predominantly right-sided motor symptoms; SD: standard deviation; STAI: State-Trait Anxiety Inventory.

^*^ *p* <.05; ^**^ *p* <.05 FDR corrected

**Table S2.3.** Intragroup comparisons (Year 1 compared to Baseline; Year 3 compared to baseline; Year 5 compared to baseline) of psychiatric scores for each PD subgroup (LPDf ; LPDm ; RPDf ; RPDm) and HC.

|  | Comparisons  Year 1 and Baseline | | | | | | Comparisons  Year 3 and Baseline | | | | | | Comparisons  Year 5 and Baseline | | | | | |
| --- | --- | --- | --- | --- | --- | --- | --- | --- | --- | --- | --- | --- | --- | --- | --- | --- | --- | --- |
|  | LPDf  (*n* = 74) | LPDm  (*n* = 105) | RPDf  (*n* = 71) | RPDm  (*n* = 163) | HCf  (*n* = 70) | HCm  (*n* = 126) | LPDf  (*n* = 74) | LPDm  (*n* = 105) | RPDf  (*n* = 71) | RPDm  (*n* = 163) | HCf  (*n* = 70) | HCm  (*n* = 126) | LPDf  (*n* = 74) | LPDm  (*n* = 105) | RPDf  (*n* = 71) | RPDm  (*n* = 163) | HCf  (*n* = 70) | HCm  (*n* = 126) |
| GDS | .204 | .474 | .508 | .217 | .821 | .216 | .114 | .161 | .626 | .052 | .357 | .652 | .099 | .581 | .854 | .004^**^ | .166 | .728 |
| STAI – State | .868 | .396 | .565 | .578 | .297 | .020^**^ | .978 | .532 | .046^*^ | .298 | .870 | .344 | .083 | .084 | .299 | .233 | .936 | .675 |
| STAI – Trait | .512 | .503 | .296 | .179 | .500 | .052 | .940 | .334 | .008^**^ | .344 | .321 | .307 | .227 | .988 | .435 | .037^*^ | .123 | .002^**^ |
| STAI – Total score | .613 | .848 | .293 | .827 | .492 | .005^**^ | .679 | .638 | .008^**^ | .756 | .947 | .130 | .060 | .360 | .190 | .075 | .460 | .021^**^ |
| Epworth Sleepiness Scale | .596 | .001^**^ | .532 | .708 | .747 | .317 | <.001^**^ | <.001^**^ | .335 | <.001^**^ | .086 | .552 | .056 | <.001^**^ | .117 | <.001^**^ | .029^*^ | .710 |
| RBD Questionnaire | .545 | .293 | .941 | .177 | .728 | .443 | .230 | .040^*^ | .170 | .053 | .003^**^ | .768 | .309 | .031^*^ | .037^*^ | <.001^**^ | .038^*^ | .836 |

***Legend*.** f: female; HC: healthy controls; GDS: Geriatric Depression Scale; LPD: patients with Parkinson’s disease (PD) who exhibit predominantly left-sided motor symptoms; m: male; RBD: REM Sleep Behavior Disorder; RPD: patients with PD who exhibit predominantly right-sided motor symptoms; SD: standard deviation; STAI: State-Trait Anxiety Inventory.

^*^ *p* <.05; ^**^ *p* <.05 FDR corrected

**A.**

**B.**

**C.**

**
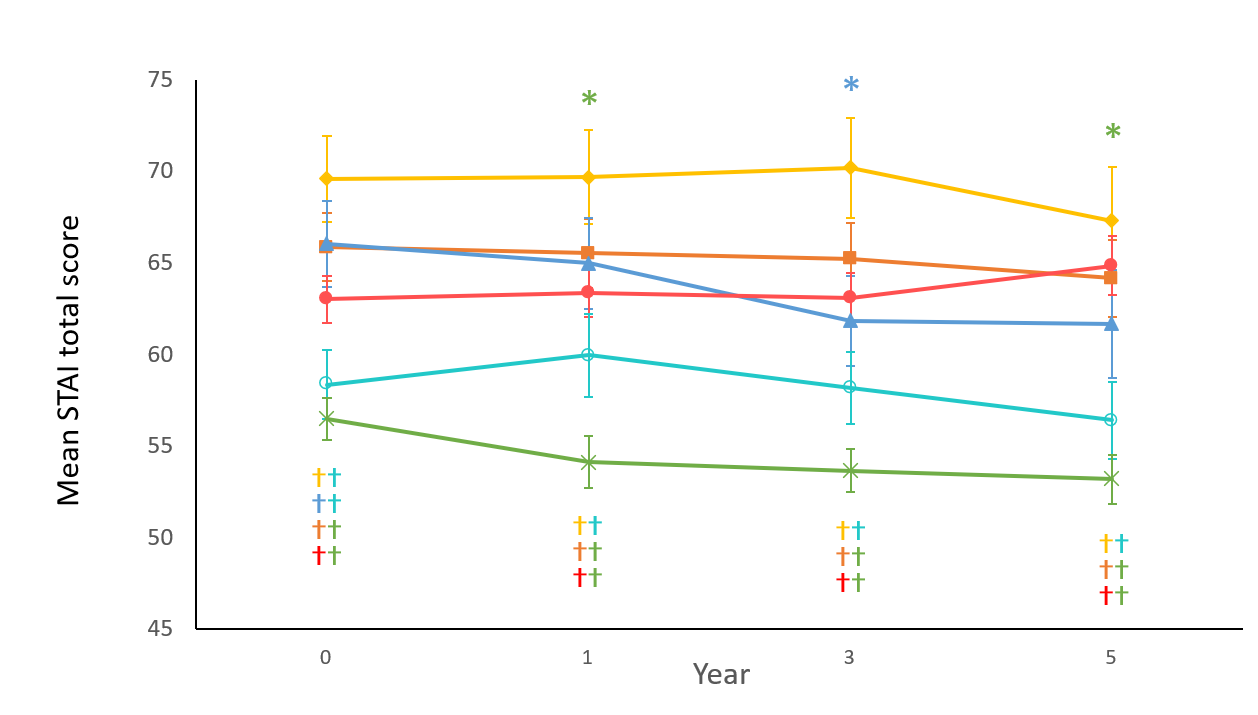

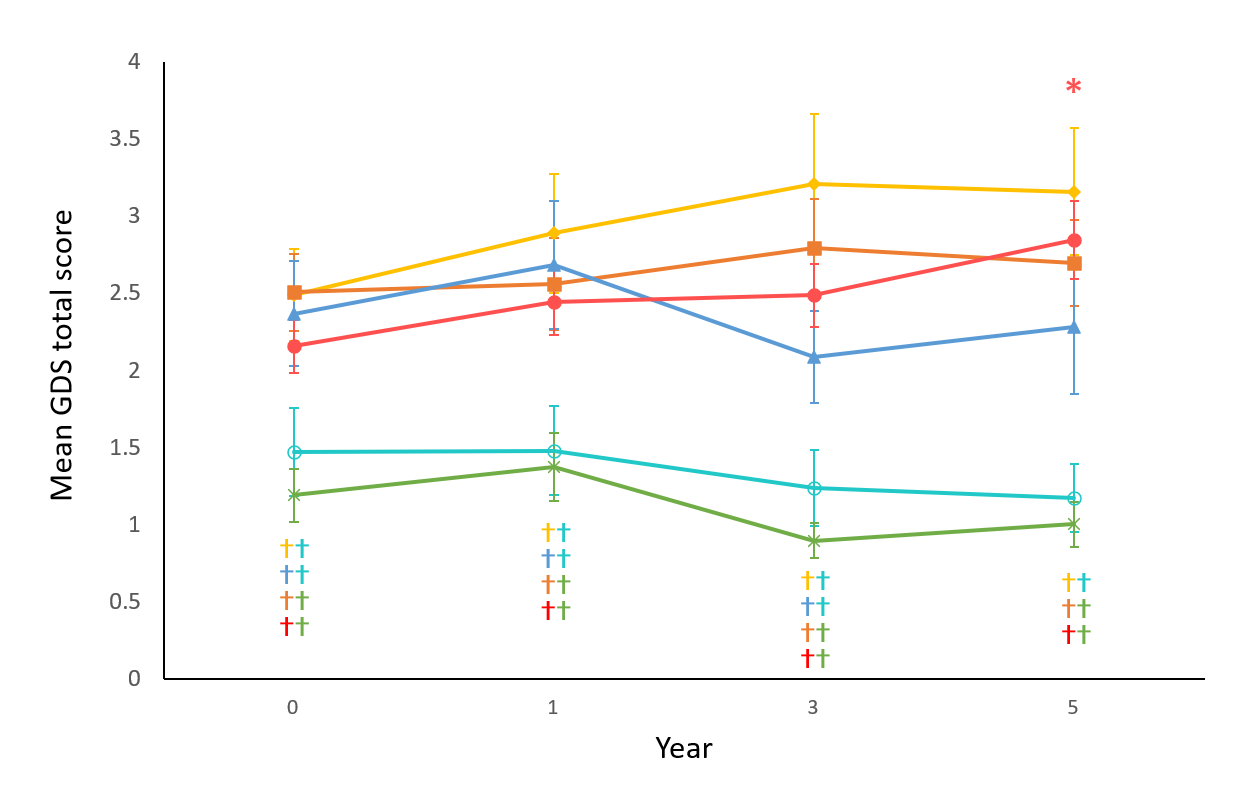
**


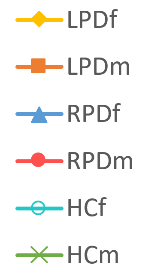


**
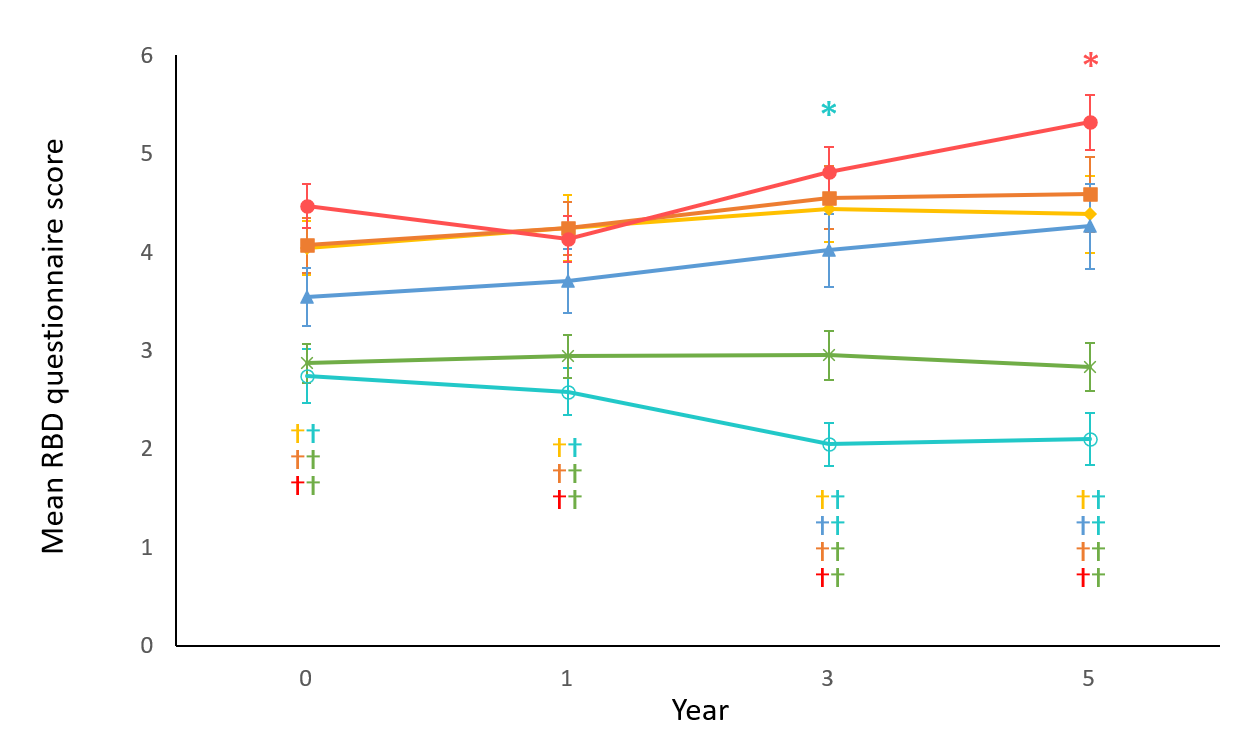

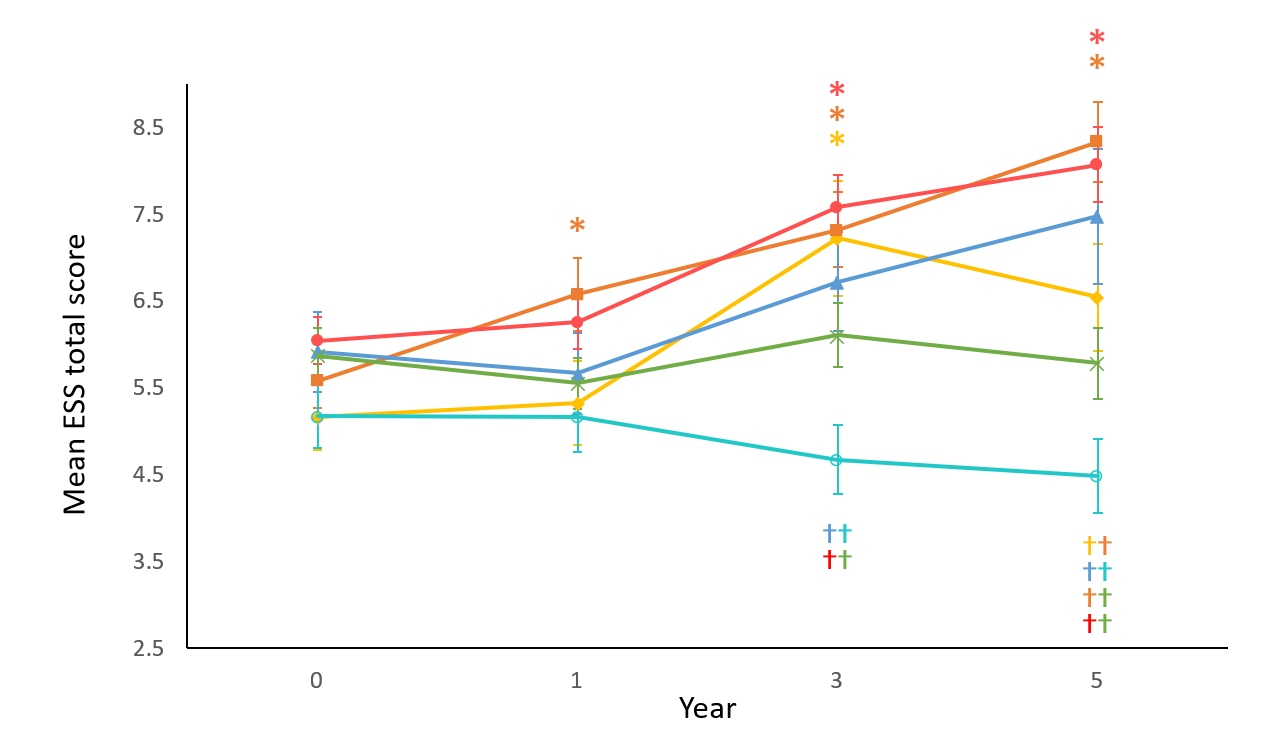
**

**Figure S2. Evolution of psychiatric scores for each PD subgroup and HC**. ESS: Epworth Sleepiness Scale; f: female; GDS: Geriatric Depression Scale; HC: healthy controls; LPD: patients with Parkinson’s disease who exhibit predominantly left-sided motor symptoms; m: male; RBD: REM Sleep Behavior Disorder; RPD: patients with PD who exhibit predominantly right-sided motor symptoms; STAI: The State-Trait Anxiety Inventory. Error bars are ± 1 SEM. * indicates a significant difference (*p* < .05) compared with baseline values. ✝✝ indicate significant differences (*p* < .05) between two subgroups at a given timepoint.

**D.**

**Table S3.1.** Motor and serum uric acid scores for each PD subgroup (LPDf ; LPDm ; RPDf ; RPDm) and HC at each timepoint.

|  | Baseline | | | | | | Year 1 | | | | | |
| --- | --- | --- | --- | --- | --- | --- | --- | --- | --- | --- | --- | --- |
|  | LPDf  (n = 74) | LPDm  (n = 105) | RPDf  (n = 71) | RPDm  (n = 163) | HCf  (n = 70) | HCm  (n = 126) | LPDf  (n = 70) | LPDm  (n = 95) | RPDf  (n = 66) | RPDm  (n = 156) | HCf  (n = 67) | HCm  (n = 118) |
| TD/PIGD classification (OFF) – orig. cat.  TD  PIGD  Undetermined | 67.12  24.66  8.22 | 70.48  18.10  11.43 | 71.83  14.08  14.08 | 72.39  16.56  11.04 | 7.14  4.29  88.57 | 16.94  4.84  78.23 | 62.07  24.14  13.79 | 66.67  22.22  11.11 | 66.04  20.75  13.21 | 70.15  20.15  9.70 | 7.46  7.46  85.07 | 19.49  5.93  74.58 |
| TD/PIGD classification (ON) – orig. cat.  TD  PIGD  Undetermined | 67.12  24.66  8.22 | 70.48  18.10  11.43 | 71.83  14.08  14.08 | 72.39  16.56  11.04 | 7.14  4.29  88.57 | 16.94  4.84  78.23 | 56.06  28.79  15.15 | 62.07  26.44  11.49 | 66.15  24.62  9.23 | 68.92  20.27  10.81 | 7.46  7.46  85.07 | 19.49  5.93  74.58 |
| MDS-UPDRS I (mean ± SD) | 6.56 (± 4.69) | 4.97 (± 3.39) | 5.65 (± 4.32) | 5.46 (± 3.97) | 3.59 (± 3.47) | 2.59 (± 2.59) | 7.16 (± 5.38) | 6.25 (± 4.43) | 7.35 (± 4.66) | 6.65 (± 4.35) | 3.67 (± 3.49) | 2.97 (± 3.02) |
| MDS-UPDRS II (mean ± SD) | 5.88 (± 4.48) | 5.57 (± 3.68) | 5.52 (± 3.56) | 6.13 (± 4.54) | 0.49 (± 0.96) | 0.45 (± 1.06) | 6.70 (± 5.01) | 7.57 (± 4.83) | 6.82 (± 4.58) | 8.01 (± 4.92) | 0.46 (± 1.28) | 0.38 (± 0.96) |
| MDS-UPDRS III (OFF) (mean ± SD) | 20.66 (± 8.29) | 22.51 (± 8.92) | 20.06 (± 8.66) | 19.99 (± 8.87) | 1.21 (± 2.35) | 1.20 (± 2.11) | 24.28 (± 10.55) | 27.22 (± 10.73) | 23.57 (± 10.48) | 24.90 (± 11.10) | 1.39 (± 2.47) | 1.86 (± 3.15) |
| MDS-UPDRS III (ON) (mean ± SD) | 20.66 (± 8.29) | 22.51 (± 8.92) | 20.06 (± 8.66) | 19.99 (± 8.87) | 1.21 (± 2.35) | 1.20 (± 2.11) | 22.46 (± 9.90) | 24.71 (± 10.71) | 22.4 (± 10.67) | 22.69 (± 10.96) | 1.39 (± 2.47) | 1.86 (± 3.15) |
| MDS-UPDRS IV score (mean ± SD) | - | - | - | - | - | - | 0.63 (± 1.37) | 0.31 (± 1.12) | 0.27 (± 0.87) | 0.41 (± 1.51) | NA | NA |
| MDS-UPDRS Total score (OFF) (mean ± SD) | 33.01 (± 13.42) | 33.06 (± 12.27) | 31.23 (± 12.58) | 31.59 (± 13.37) | 5.29 (± 5.10) | 4.15 (± 3.92) | 37.82 (± 16.27) | 40.73 (± 14.98) | 38.02 (± 15.95) | 39.83 (± 16.35) | 5.52 (± 5.41) | 5.21 (± 5.03) |
| MDS-UPDRS Total score (ON) (mean ± SD) | 33.01 (± 13.42) | 33.06 (± 12.27) | 31.23 (± 12.58) | 31.59 (± 13.37) | 5.29 (± 5.10) | 4.15 (± 3.92) | 36.60 (± 16.54) | 38.59 (± 13.98) | 36.52 (± 16.09) | 36.85 (± 15.36) | 5.52 (± 5.41) | 5.21 (± 5.03) |
| H&Y Stage (OFF Stage)  stage 0  stage 1  stage 2  stage 3  stage 4  stage 5 | 0.00  44.59  54.05  1.35  -  - | 0.00  35.24  63.81  0.95  -  - | 0.00  46.48  53.52  0.00  -  - | 0.00  50.31  49.69  0.00  -  - | 100.00  0.00  0.00  0.00  -  - | 98.40  1.60  0.00  0.00  -  - | 0.00  30.51  64.41  3.39  1.69  - | 0.00  24.69  72.84  2.47  0.00  - | 0.00  33.96  60.38  5.66  0.00  - | 0.74  30.37  65.19  3.70  0.00  - | 98.51  1.49  0.00  0.00  0.00  - | 94.92  1.69  3.39  0.00  0.00  - |
| H&Y Stage (ON Stage)  stage 0  stage 1  stage 2  stage 3  stage 4  stage 5 | 0.00  44.59  54.05  1.35  -  - | 0.00  35.24  63.81  0.95  -  ­- | 0.00  46.48  53.52  0.00  -  - | 0.00  50.31  49.69  0.00  -  - | 100.00  0.00  0.00  0.00  -  - | 98.40  1.60  0.00  0.00  -  - | 0.00  31.34  64.18  2.99  1.49  - | 0.00  24.14  75.86  0.00  0.00  - | 0.00  32.31  63.08  4.62  0.00  - | 0.67  28.86  68.46  2.01  0.00  - | 98.51  1.49  0.00  0.00  0.00  - | 94.92  1.69  3.39  0.00  0.00  - |
| LEDD (mean ± SD) | - | - | - | - | - | - | 312.42 (± 225.73) | 274.97 (± 232.69) | 301.17 (± 246.50) | 296.37 (± 182.57) | NA | NA |
| Serum Uric Acid (mean ± SD) | 259.93 (± 52.42) | 346.38 (± 73.00) | 252.99 (± 52.05) | 349.59 (± 69.93) | 270.74 (± 56.75) | 352.08 (± 72.30) | 259.81 (± 57.20) | 334.77 (± 68.21) | 244.28 (± 48.58) | 345.51 (± 67.41) | 269.27 (± 57.00) | 349.59 (± 66.86) |
|  | Year 3 | | | | | | Year 5 | | | | | |
|  | LPDf  (n = 67) | LPDm  (n = 87) | RPDf  (n = 59) | RPDm  (n = 144) | HCf  (n = 64) | HCm  (n = 103) | LPDf  (*n* = 59) | LPDm  (*n* = 75) | RPDf  (*n* = 46) | RPDm  (*n* = 129) | HCf  (*n* = 58) | HCm  (*n* = 96) |
| TD/PIGD classification (OFF) – orig. cat.  TD  PIGD  Undetermined | 70.21  21.28  8.51 | 58.82  30.88  10.29 | 59.46  32.43  8.11 | 64.42  26.92  8.65 | 10.94  10.94  78.13 | 19.61  7.84  72.55 | 52.38  33.33  14.29 | 52.63  31.58  15.79 | 51.52  24.24  24.24 | 67.37  27.37  5.26 | 12.07  13.79  74.14 | 19.79  10.42  69.79 |
| TD/PIGD classification (ON) – orig. cat.  TD  PIGD  Undetermined | 58.06  29.03  12.90 | 65.38  26.92  7.69 | 56.14  31.58  12.28 | 62.32  28.99  8.70 | 10.94  10.94  78.13 | 19.61  7.84  72.55 | 32.14  53.57  14.29 | 50.00  39.19  10.81 | 38.10  38.10  23.81 | 50.41  38.84  10.74 | 12.07  13.79  74.14 | 19.79  10.42  69.79 |
| MDS-UPDRS I (mean ± SD) | 9.00 (± 6.49) | 7.46 (± 5.23) | 7.78 (± 4.55) | 8.56 (± 5.25) | 3.73 (± 3.45) | 3.16 (± 2.70) | 9.61 (± 6.73) | 9.19 (± 6.39) | 8.37 (± 5.78) | 9.51 (± 6.04) | 3.59 (± 3.17) | 3.45 (± 3.03) |
| MDS-UPDRS II (mean ± SD) | 7.78 (± 6.32) | 9.08 (± 5.31) | 7.54 (± 5.39) | 9.72 (± 5.44) | 0.42 (± 0.99) | 0.65 (± 1.20) | 8.75 (± 7.23) | 10.05 (± 6.35) | 6.93 (± 4.63) | 11.69 (± 6.81) | 0.59 (± 1.43) | 0.90 (± 1.72) |
| MDS-UPDRS III (OFF) (mean ± SD) | 27.85 (± 12.56) | 30.6 (± 11.71) | 27.86 (± 13.53) | 29.55 (± 12.00) | 1.77 (± 3.58) | 1.49 (± 3.37) | 30.74 (± 15.09) | 31.32 (± 13.49) | 26.76 (± 10.73) | 32.81 (± 10.75) | 1.88 (± 3.02) | 2.93 (± 4.47) |
| MDS-UPDRS III (ON) (mean ± SD) | 21.76 (± 12.79) | 25.14 (± 11.94) | 23.77 (± 13.28) | 24.28 (± 11.57) | 1.77 (± 3.58) | 1.49 (± 3.37) | 22.39 (± 15.03) | 24.99 (± 13.76) | 19.98 (± 10.29) | 26.59 (± 12.51) | 1.88 (± 3.02) | 2.93 (± 4.47) |
| MDS-UPDRS IV score (mean ± SD) | 1.66 (± 2.51) | 0.84 (± 1.77) | 0.69 (± 1.53) | 0.68 (± 1.63) | 0.00 (± 0.00) | 0.00 (± 0.00) | 3.02 (± 3.86) | 2.39 (± 2.62) | 1.81 (± 2.92) | 1.64 (± 2.54) | 0.00 (± 0.00) | 0.00 (± 0.00) |
| MDS-UPDRS Total score (OFF) (mean ± SD) | 43.96 (± 21.33) | 46.71 (± 16.81) | 43.46 (± 20.29) | 47.26 (± 17.86) | 5.92 (± 5.80) | 5.29 (± 4.84) | 48.88 (± 22.74) | 49.3 (± 21.00) | 42.09 (± 14.93) | 53.15 (± 17.37) | 6.05 (± 5.27) | 7.27 (± 6.94) |
| MDS-UPDRS Total score (ON) (mean ± SD) | 38.45 (± 21.57) | 41.19 (± 16.37) | 39.02 (± 19.08) | 42.52 (± 17.70) | 5.92 (± 5.80) | 5.29 (± 4.84) | 41.16 (± 24.33) | 44.36 (± 20.69) | 35.48 (± 15.00) | 47.56 (± 20.99) | 6.05 (± 5.27) | 7.27 (± 6.94) |
| H&Y Stage (OFF Stage)  stage 0  stage 1  stage 2  stage 3  stage 4  stage 5 | 0.00  12.77  82.98  2.13  0.00  2.13 | 0.00  17.65  76.47  5.88  0.00  0.00 | 0.00  27.03  54.05  18.92  0.00  0.00 | 0.00  17.31  75.00  5.77  1.92  0.00 | 100.00  0.00  0.00  0.00  0.00  0.00 | 98.04  0.98  0.98  0.00  0.00  0.00 | 0.00  9.30  79.07  9.30  -  2.33 | 0.00  7.02  87.72  3.51  -  1.75 | 0.00  18.18  78.79  3.03  -  0.00 | 0.00  7.37  88.42  4.21  -  0.00 | 98.28  1.72  0.00  0.00  -  0.00 | 98.96  1.04  0.00  0.00  -  0.00 |
| H&Y Stage (ON Stage)  stage 0  stage 1  stage 2  stage 3  stage 4  stage 5 | 1.61  14.52  79.03  3.23  1.61  - | 0.00  29.49  66.67  3.85  0.00  - | 0.00  24.56  68.42  7.02  0.00  - | 0.00  22.46  71.74  5.07  0.72  - | 100.00  0.00  0.00  0.00  0.00  - | 98.04  0.98  0.98  0.00  0.00  - | 0.00  10.53  78.95  7.02  3.51  0.00 | 0.00  14.86  78.38  5.41  0.00  1.35 | 0.00  28.57  71.43  0.00  0.00  0.00 | 0.00  11.57  82.64  4.13  1.65  0.00 | 98.28  1.72  0.00  0.00  0.00  0.00 | 98.96  1.04  0.00  0.00  0.00  0.00 |
| LEDD (mean ± SD) | 457.35 (± 304.87) | 517.77 (± 319.05) | 483.58 (± 758.70) | 496.40 (± 349.35) | NA | NA | 803.64 (± 1343.55) | 790.96 (± 711.60) | 548.41 (± 467.45) | 657.26 (± 296.80) | NA | NA |
| Serum Uric Acid (mean ± SD) | 261.75 (± 65.67) | 330.55 (± 69.56) | 262.11 (± 52.58) | 340.76 (± 75.16) | 278.56 (± 62.26) | 350.89 (± 81.32) | 264.28 (± 68) | 333.46 (± 74.41) | 252.20 (± 48.81) | 336.86 (± 76.79) | 276.71 (± 55.23) | 352.46 (± 71.86) |

***Legend*.** f: female; H&Y: Hoehn and Yahr scale; HC: healthy controls; LEDD: total Levodopa Equivalent Daily Dose; LPD: patients with Parkinson’s disease (PD) who exhibit predominantly left-sided motor symptoms; m: male; PIGD: postural instability and gait difficulty; RPD: patients with PD who exhibit predominantly right-sided motor symptoms; TD: tremor dominant; MDS-UPDRS: Movement Disorders Society Unified Parkinson's Disease Rating Scale.

**Table S3.2.** Intergroup comparisons at each timepoint of motor and serum uric acid outcomes for each PD subgroup (LPDf ; LPDm ; RPDf ; RPDm) and HC.

|  | Baseline  K-W and M-W/Khi2 Bonferroni correction | | | | | | | | | Year 1  K-W and M-W/Khi2 Bonferroni correction | | | | | | | | |
| --- | --- | --- | --- | --- | --- | --- | --- | --- | --- | --- | --- | --- | --- | --- | --- | --- | --- | --- |
|  | K-W/ Khi^2^ | LPDf vs LPDm | RPDf vs RPDm | LPDf vs RPDf | LPDm vs RPDm | LPDf vs HCf | LPDm vs HCm | RPDf vs HCf | RPDm vs HCm | K-W/ Khi^2^ | LPDf vs LPDm | RPDf vs RPDm | LPDf vs RPDf | LPDm vs RPDm | LPDf vs HCf | LPDm vs HCm | RPDf vs HCf | RPDm vs HCm |
| TD/PIGD classification (OFF) – orig. cat. | < .001^*^ | .496 | .749 | .192 | .938 | <.001^**^ | <.001^**^ | <.001^**^ | <.001^**^ | - | .834 | .765 | .898 | .865 | <.001^**^ | <.001^**^ | <.001^**^ | <.001^**^ |
| TD/PIGD classification (ON) – orig. cat. | < .001^*^ | .496 | .749 | .192 | .938 | <.001^**^ | <.001^**^ | <.001^**^ | <.001^**^ | - | .709 | .758 | .427 | .513 | <.001^**^ | <.001^**^ | <.001^**^ | <.001^**^ |
| MDS-UPDRS I | < .001^*^ | .030^*^ | .904 | .193 | .478 | <.001^**^ | <.001^**^ | .002^**^ | <.001^**^ | < .001^*^ | .442 | .329 | .489 | .420 | <.001^**^ | <.001^**^ | <.001^**^ | <.001^**^ |
| MDS-UPDRS II | < .001^*^ | .975 | .741 | .984 | .667 | <.001^**^ | <.001^**^ | <.001^**^ | <.001^**^ | < .001^*^ | .142 | .076 | .634 | .555 | <.001^**^ | <.001^**^ | <.001^**^ | <.001^**^ |
| MDS-UPDRS III (OFF) | < .001^*^ | .170 | .852 | .558 | .012^*^ | <.001^**^ | <.001^**^ | <.001^**^ | <.001^**^ | < .001^*^ | .076 | .565 | .898 | .117 | <.001^**^ | <.001^**^ | <.001^**^ | <.001^**^ |
| MDS-UPDRS III (ON) | < .001^*^ | .170 | .852 | .558 | .012^*^ | <.001^**^ | <.001^**^ | <.001^**^ | <.001^**^ | < .001^*^ | .143 | .830 | .993 | .128 | <.001^**^ | <.001^**^ | <.001^**^ | <.001^**^ |
| MDS-UPDRS IV score | - | - | - | - | - | - | - | - | - | .312 | - | - | - | - | - | - | - | - |
| MDS-UPDRS Total score (OFF) | < .001^*^ | .991 | .857 | .344 | .280 | <.001^**^ | <.001^**^ | <.001^**^ | <.001^**^ | < .001^*^ | .130 | .641 | .749 | .387 | <.001^**^ | <.001^**^ | <.001^**^ | <.001^**^ |
| MDS-UPDRS Total score (ON) | < .001^*^ | .991 | .857 | .344 | .280 | <.001^**^ | <.001^**^ | <.001^**^ | <.001^**^ | < .001^*^ | .130 | .855 | .748 | .209 | <.001^**^ | <.001^**^ | <.001^**^ | <.001^**^ |
| H&Y Stage (OFF Stage) | < .001^*^ | .422 | .590 | .610 | .028 | <.001^**^ | <.001^**^ | <.001^**^ | <.001^**^ | - | .522 | .794 | .706 | .604 | <.001^**^ | <.001^**^ | <.001^**^ | <.001^**^ |
| H&Y Stage (ON Stage) | < .001^*^ | .422 | .590 | .610 | .028 | <.001^**^ | <.001^**^ | <.001^**^ | <.001^**^ | - | .148 | .592 | .749 | .360 | <.001^**^ | <.001^**^ | <.001^**^ | <.001^**^ |
| LEDD | - | - | - | - | - | - | - | - | - | .243 | - | - | - | - | - | - | - | - |
| Serum Uric Acid | < .001^*^ | <.001^**^ | <.001^**^ | .468 | .826 | .108 | .600 | .039^*^ | .630 | < .001^*^ | <.001^**^ | <.001^**^ | .163 | .142 | .212 | .057^*^ | .003^**^ | .698 |
|  | Year 3  K-W and M-W/Khi2 Bonferroni correction | | | | | | | | | Year 5  K-W and M-W/Khi2 Bonferroni correction | | | | | | | | |
|  | K-W/ Khi^2^ | LPDf vs LPDm | RPDf vs RPDm | LPDf vs RPDf | LPDm vs RPDm | LPDf vs HCf | LPDm vs HCm | RPDf vs HCf | RPDm vs HCm | K-W/ Khi^2^ | LPDf vs LPDm | RPDf vs RPDm | LPDf vs RPDf | LPDm vs RPDm | LPDf vs HCf | LPDm vs HCm | RPDf vs HCf | RPDm vs HCm |
| TD/PIGD classification (OFF) – orig. cat. | - | .447 | .815 | .508 | .759 | <.001^**^ | <.001^**^ | <.001^**^ | <.001^**^ | - | .971 | .008^*^ | .471 | .056 | <.001^**^ | <.001^**^ | <.001^**^ | <.001^**^ |
| TD/PIGD classification (ON) – orig. cat. | - | .524 | .645 | .955 | .901 | <.001^**^ | <.001^**^ | <.001^**^ | <.001^**^ | - | .124 | .092 | .265 | .998 | <.001^**^ | <.001^**^ | <.001^**^ | <.001^**^ |
| MDS-UPDRS I | < .001^*^ | .187 | .468 | .647 | .078 | <.001^**^ | <.001^**^ | <.001^**^ | <.001^**^ | < .001^*^ | .779 | .243 | .406 | .623 | <.001^**^ | <.001^**^ | <.001^**^ | <.001^**^ |
| MDS-UPDRS II | < .001^*^ | .040^*^ | .005^**^ | .895 | .443 | <.001^**^ | <.001^**^ | <.001^**^ | <.001^**^ | < .001^*^ | .104 | < 001^**^ | .344 | .075 | <.001^**^ | <.001^**^ | <.001^**^ | <.001^**^ |
| MDS-UPDRS III (OFF) | < .001^*^ | .098 | .419 | .978 | .567 | <.001^**^ | <.001^**^ | <.001^**^ | <.001^**^ | < .001^*^ | .463 | .007^**^ | .575 | .472 | <.001^**^ | <.001^**^ | <.001^**^ | <.001^**^ |
| MDS-UPDRS III (ON) | < .001^*^ | .066 | .610 | .357 | .568 | <.001^**^ | <.001^**^ | <.001^**^ | <.001^**^ | < .001^*^ | .178 | .004** | .777 | .286 | <.001^**^ | <.001^**^ | <.001^**^ | <.001^**^ |
| MDS-UPDRS IV score | .019^*^ | .049^*^ | .607 | .027^*^ | .209 | - | - | - | - | .002^*^ | .724 | .921 | .076 | .014^**^ | - | - | - | - |
| MDS-UPDRS Total score (OFF) | < .001^*^ | .158 | .289 | .836 | .894 | <.001^**^ | <.001^**^ | <.001^**^ | <.001^**^ | < .001^*^ | .653 | .002^**^ | .402 | .122 | <.001^**^ | <.001^**^ | <.001^**^ | <.001^**^ |
| MDS-UPDRS Total score (ON) | < .001^*^ | .092 | .182 | .549 | .590 | <.001^**^ | <.001^**^ | <.001^**^ | <.001^**^ | < .001^*^ | .266 | .001^**^ | .413 | .192 | <.001^**^ | <.001^**^ | <.001^**^ | <.001^**^ |
| H&Y Stage (OFF Stage) | - | .404 | .031^*^ | .009^**^ | .724 | <.001^**^ | <.001^**^ | <.001^**^ | <.001^**^ | - | .617 | .205 | .391 | .633 | <.001^**^ | <.001^**^ | <.001^**^ | <.001^**^ |
| H&Y Stage (ON Stage) | - | .154 | .844 | .321 | .595 | <.001^**^ | <.001^**^ | <.001^**^ | <.001^**^ | - | .410 | .036^*^ | .030^*^ | .476 | <.001^**^ | <.001^**^ | <.001^**^ | <.001^**^ |
| LEDD | .009^*^ | .175 | .011^**^ | .172 | .534 | - | - | - | - | .007^*^ | .265 | .002^**^ | .047^*^ | .404 | - | - | - | - |
| Serum Uric Acid | < .001^*^ | <.001^**^ | <.001^**^ | .718 | .383 | .043^*^ | .063 | .095 | .198 | < .001^*^ | <.001^**^ | <.001^**^ | .437 | .666 | .156 | .056 | .031^*^ | .066 |

***Legend*.** f: female; H&Y: Hoehn and Yahr scale; HC: healthy controls; LEDD: total Levodopa Equivalent Daily Dose; LPD: patients with Parkinson’s disease (PD) who exhibit predominantly left-sided motor symptoms; m: male; PIGD: postural instability and gait difficulty; RPD: patients with PD who exhibit predominantly right-sided motor symptoms; TD: tremor dominant; MDS-UPDRS: Movement Disorders Society Unified Parkinson's Disease Rating Scale.

^*^ *p* <.05; ^**^ *p* <.05 FDR corrected

**Table S3.3.** Intragroup comparisons (Year 1 compared to Baseline; Year 3 compared to baseline; Year 5 compared to baseline) of motor scores and serum uric acid for each PD subgroup (LPDf ; LPDm ; RPDf ; RPDm) and HC.

|  | Comparisons  Year 1 and Baseline | | | | | | Comparisons  Year 3 and Baseline | | | | | | Comparisons  Year 5 and Baseline | | | | | |
| --- | --- | --- | --- | --- | --- | --- | --- | --- | --- | --- | --- | --- | --- | --- | --- | --- | --- | --- |
|  | LPDf  (*n* = 74) | LPDm  (*n* = 105) | RPDf  (*n* = 71) | RPDm  (*n* = 163) | HCf  (*n* = 70) | HCm  (*n* = 126) | LPDf  (*n* = 74) | LPDm  (*n* = 105) | RPDf  (*n* = 71) | RPDm  (*n* = 163) | HCf  (*n* = 70) | HCm  (*n* = 126) | LPDf  (*n* = 74) | LPDm  (*n* = 105) | RPDf  (*n* = 71) | RPDm  (*n* = 163) | HCf  (*n* = 70) | HCm  (*n* = 126) |
| MDS-UPDRS I | .078 | .001^**^ | <.001^**^ | <.001^**^ | .590 | .111 | <.001^**^ | <.001^**^ | <.001^**^ | <.001^**^ | .936 | .016^*^ | <.001^**^ | <.001^**^ | <.001^**^ | <.001^**^ | .621 | .003^**^ |
| MDS-UPDRS II | .019 | <.001^**^ | .042^*^ | <.001^**^ | .354 | .779 | .001^**^ | <.001^**^ | .001 | <.001^**^ | .639 | .027^*^ | <.001^**^ | <.001^**^ | .003^**^ | <.001^**^ | .409 | .002^**^ |
| MDS-UPDRS III (OFF) | <.001^**^ | <.001^**^ | .001^**^ | <.001^**^ | .181 | .003^**^ | <.001^**^ | <.001^**^ | <.001^**^ | <.001^**^ | .087 | .550 | <.001^**^ | <.001^**^ | <.001^**^ | <.001^**^ | .007^**^ | <.001^**^ |
| MDS-UPDRS III (ON) | .023^*^ | .036^*^ | .061 | <.001^**^ | .181 | .003^**^ | .695 | .004^**^ | .044^*^ | <.001^**^ | .087 | .550 | .538 | .017^*^ | .326 | <.001^**^ | .007^**^ | <.001^**^ |
| MDS-UPDRS IV score | - | - | - | - | - | - | .001^**^ | .030^*^ | .441 | .004^**^ | - | - | <.001^**^ | <.001^**^ | .013^*^ | <.001^**^ | - | - |
| MDS-UPDRS Total score (OFF) | <.001^**^ | <.001^**^ | <.001^**^ | <.001^**^ | .796 | .008^**^ | <.001^**^ | <.001^**^ | <.001^**^ | <.001^**^ | .279 | .007^**^ | <.001^**^ | <.001^**^ | <.001^**^ | <.001^**^ | .085 | <.001^**^ |
| MDS-UPDRS Total score (ON) | .003^**^ | <.001^**^ | .009^**^ | <.001^**^ | .796 | .008^**^ | .073 | <.001^**^ | .001^**^ | <.001^**^ | .279 | .007^**^ | .007^**^ | <.001^**^ | .001^**^ | <.001^**^ | .085 | <.001^**^ |
| LEDD^¥^ | - | - | - | - | - | - | <.001^**^ | <.001^**^ | <.001^**^ | <.001^**^ | - | - | <.001^**^ | <.001^**^ | <.001^**^ | <.001^**^ | - | - |
| Serum Uric Acid | .449 | .024^**^ | .201 | .155 | .895 | .841 | 1.00 | .029^**^ | .602 | .096 | .373 | .890 | .374 | .039^*^ | .885 | .054 | .029^*^ | .840 |

***Legend*.** f: female; H&Y: Hoehn and Yahr scale; HC: healthy controls; LEDD: total Levodopa Equivalent Daily Dose; LPD: patients with Parkinson’s disease (PD) who exhibit predominantly left-sided motor symptoms; m: male; PIGD: postural instability and gait difficulty; RPD: patients with PD who exhibit predominantly right-sided motor symptoms; TD: tremor dominant; MDS-UPDRS: Movement Disorders Society Unified Parkinson's Disease Rating Scale. ^¥^ For the LEDD variable, Year 1 was taken as baseline for intragroup comparison given absence of treatment at the beginning of the study (comparisons were carried out as follows: Year 1 compared to Year 3; Year 1 compared to Year 5).

^*^ *p* <.05; ^**^ *p* <.05 FDR corrected

**
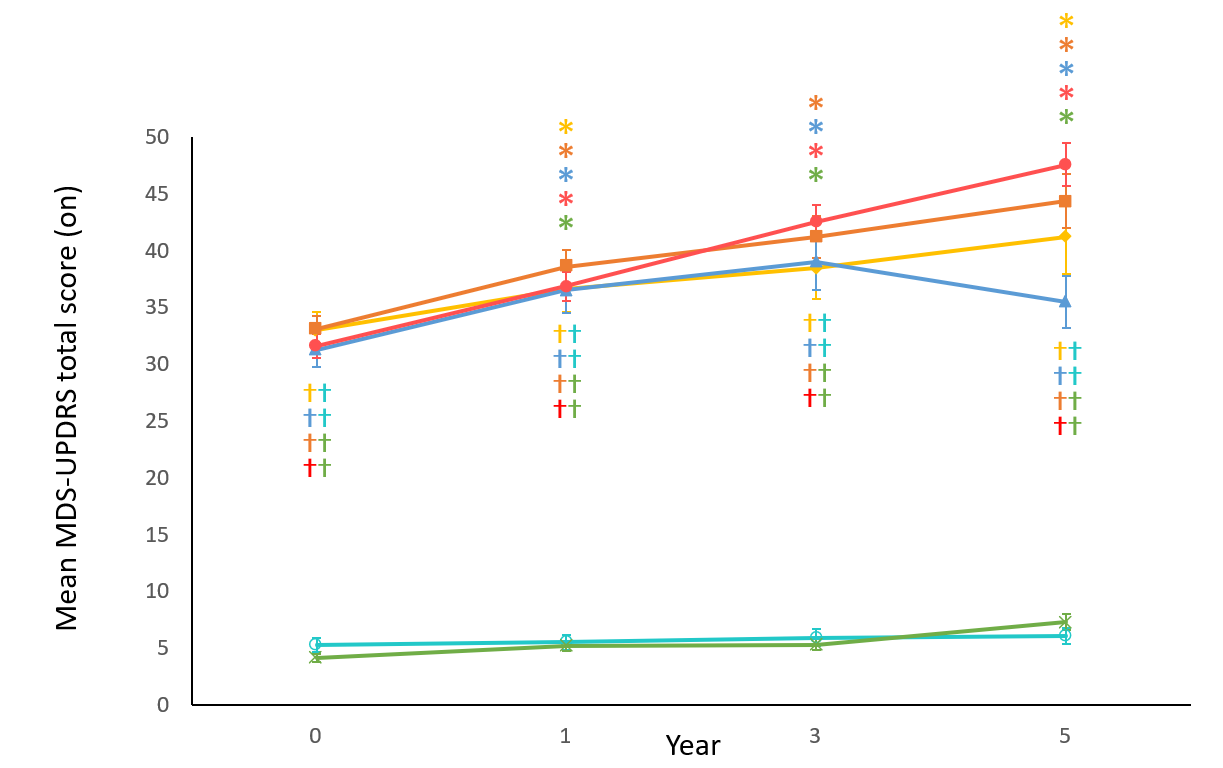
**
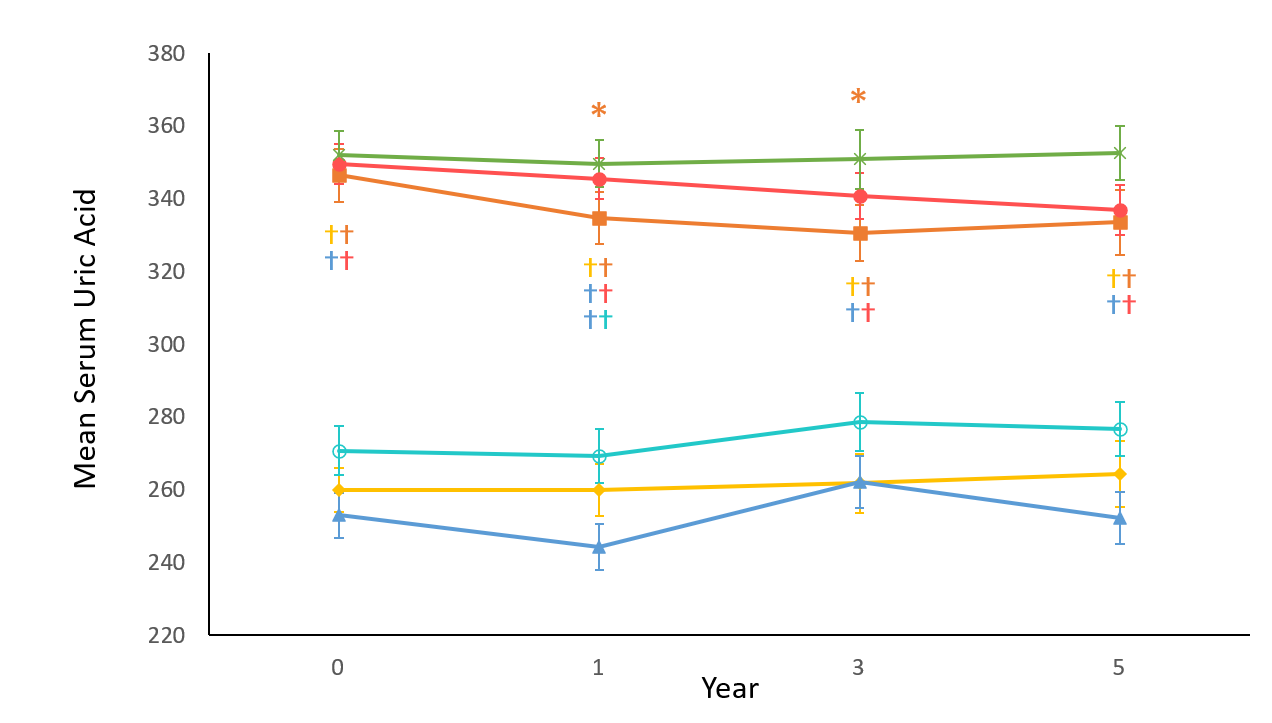
**
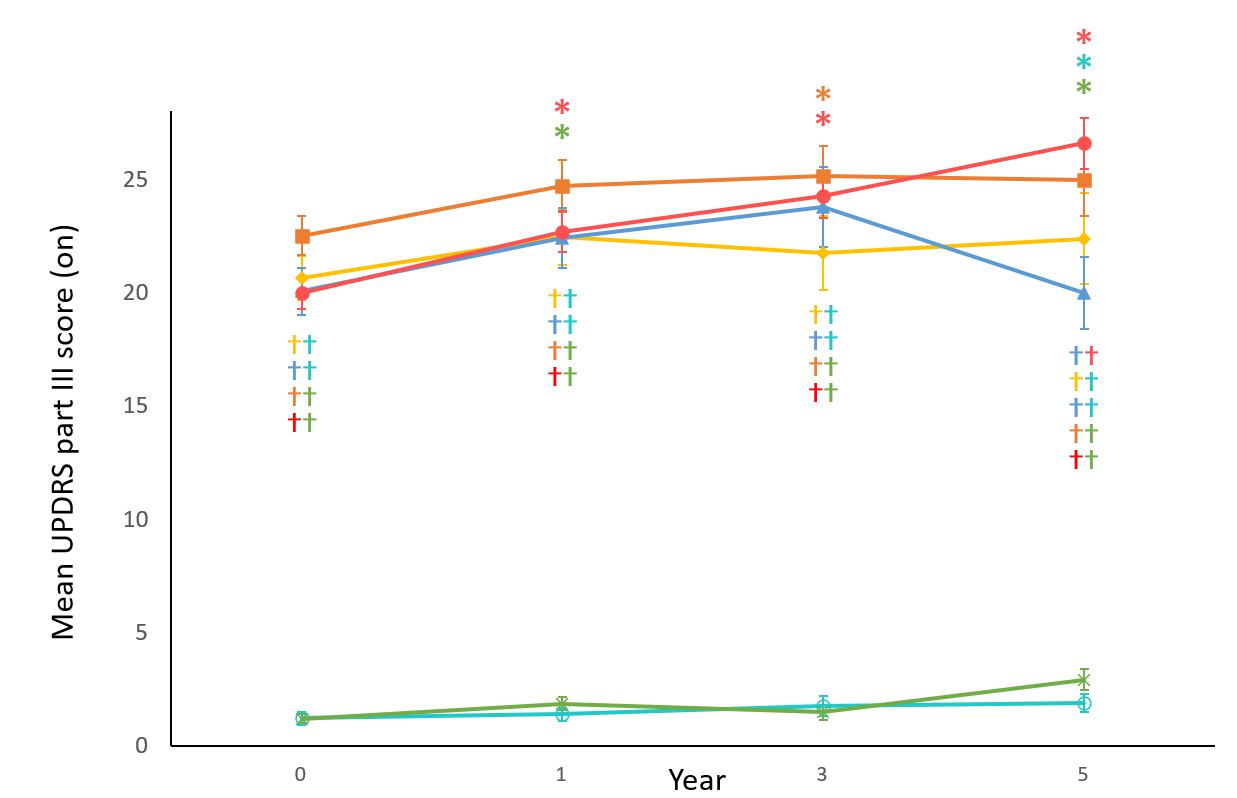
**

**B.**

**A.**

**C.**


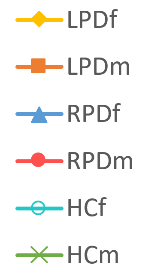


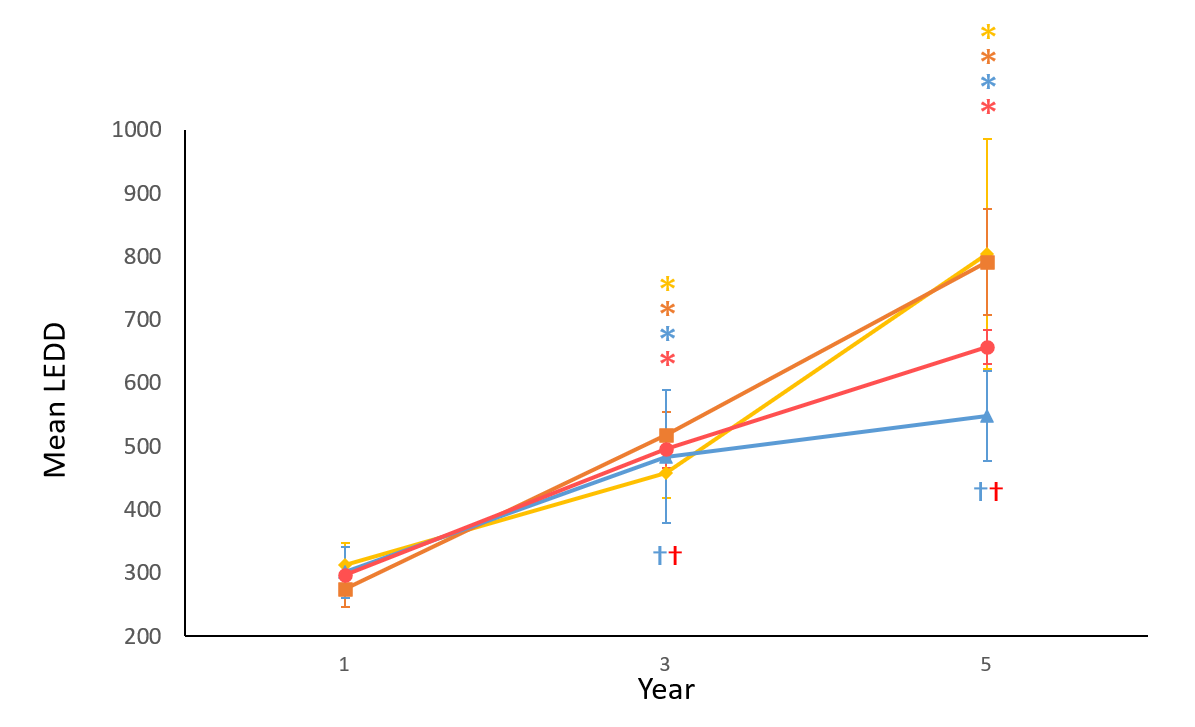


**D.**

**Figure S3. Evolution of motor and serum uric acid outcomes for each PD subgroup and HC**. f: female; HC: healthy controls; LEDD: total Levodopa Equivalent Daily Dose; LPD: patients with Parkinson’s disease (PD) who exhibit predominantly left-sided motor symptoms; m: male; RPD: patients with PD who exhibit predominantly right-sided motor symptoms; MDS-UPDRS: Movement Disorders Society Unified Parkinson's Disease Rating Scale. Error bars are ± 1 SEM. * indicates a significant difference (*p* < .05) compared with baseline values. ✝✝ indicate significant differences (*p* < .05) between two subgroups at a given timepoint.

**Table S4.** Correlation between the Epworth sleepiness scale scores and the Symbol Digit Modalities Test scores for the whole sample and each subgroup.

|  | r^2^ | *p* |
| --- | --- | --- |
| Whole sample  (n = 609) | -.123** | <.001 |
| LPDf  (n = 74) | -.051 | .309 |
| LPDm  (n = 105) | -.132** | .002 |
| RPDf  (n = 71) | -.154** | .004 |
| RPDm  (n = 163) | -.219** | <.001 |
| HCf  (n = 70) | .239** | <.001 |
| HCm  (n = 126) | .052 | .183 |

***Legend*.** f: female; HC: healthy controls; LPD: patients with Parkinson’s disease (PD) who exhibit predominantly left-sided motor symptoms; m: male; RPD: patients with PD who exhibit predominantly right-sided motor symptoms. r^2^: Pearson correlation coefficient.

^**^*p* <.001 (FDR corrected)
